# Multinuclear NMR and MRI Beyond Proton Imaging: Principles, Contrast Mechanisms, and Applications in Materials and Biomedicine

**DOI:** 10.3390/ijms27104384

**Published:** 2026-05-14

**Authors:** Dorota Bartusik-Aebisher, Klaudia Dynarowicz, Barbara Smolak, Rostyslav Marunych, Wiesław Guz, David Aebisher

**Affiliations:** 1 Department of Biochemistry and General Chemistry, Faculty of Medicine, University of Rzeszów, 35-310 Rzeszów, Poland; kdynarowicz@ur.edu.pl; 2 Department of Diagnostic Imaging and Nuclear Medicine, Faculty of Medicine, University of Rzeszów, 35-310 Rzeszów, Poland; bsmolak@ur.edu.pl (B.S.); wguz@ur.edu.pl (W.G.); 3 Doctoral School, Faculty of Medicine, Collegium Medicum, University of Rzeszów, 35-310 Rzeszów, Poland; rostyslavm@dokt.ur.edu.pl; 4 Department of Photomedicine and Physical Chemistry, Faculty of Medicine, University of Rzeszów, 35-310 Rzeszów, Poland

**Keywords:** multinuclear MRI, NMR spectroscopy, proton MRI, molecular imaging, contrast mechanisms, RF coils, signal-to-noise ratio, biomaterials

## Abstract

Magnetic resonance techniques have evolved beyond conventional proton-based imaging, enabling access to a broader range of nuclei that provide complementary structural, functional, and molecular information. This review presents a comprehensive overview of multinuclear NMR and MRI in solid and soft materials as well as in biomedical applications, with particular emphasis on ^1^H, ^13^C, ^31^P, ^23^Na, and ^19^F nuclei. Proton-based methods remain the foundation of magnetic resonance due to their high sensitivity and widespread applicability, offering insights into molecular mobility, hydration, and microstructural heterogeneity. In contrast, heteronuclear approaches enable more specific characterization of chemical structure (^13^C), phosphorus-containing functional groups and membranes (^31^P), ionic homeostasis and transport (^23^Na), and exogenous tracers with negligible biological background (^19^F). Together, these techniques extend magnetic resonance from primarily anatomical imaging toward functional, metabolic, and molecular-level analysis. The review further discusses key hardware aspects, including magnetic field strength and radiofrequency coil design, highlighting the trade-offs between low- and high-field systems and the growing importance of multinuclear coil architectures. For example, because ^1^H, ^23^Na, ^31^P, and ^19^F resonate at different Larmor frequencies, multinuclear experiments require dedicated or multi-tuned RF coils that balance sensitivity, field homogeneity, and decoupling between channels. Mechanisms of contrast generation are examined in detail, distinguishing between endogenous sources—such as water, ions, and metabolites—and exogenous contrast agents, including gadolinium-, manganese-, and fluorine-based compounds, as well as targeted and theranostic platforms. A comparative framework of endogenous and exogenous signals is presented, emphasizing their complementary roles in balancing safety, specificity, and sensitivity. Finally, the opportunities and challenges of multinuclear magnetic resonance are critically evaluated, including limitations in sensitivity, signal-to-noise ratio, data interpretation in heterogeneous systems, and technical complexity. Emerging directions such as ultrahigh-field imaging, advanced RF technologies, hyperpolarization, and artificial intelligence-assisted reconstruction are discussed as key drivers for future development. Overall, multinuclear NMR and MRI represent a powerful and expanding toolbox for probing complex material and biological systems, with the potential to significantly enhance diagnostic capabilities and deepen our understanding of structure–function relationships across multiple scales.

## 1. Introduction

Magnetic resonance, including nuclear magnetic resonance (NMR) spectroscopy and magnetic resonance imaging (MRI), is one of the most important non-destructive analytical techniques used in chemistry and materials science. It enables the investigation of chemical composition, local atomic environments, and molecular dynamics across a wide range of temporal and spatial scales. Unlike many existing reviews that focus on either solid-state NMR methodology, selected X-nuclei, specific contrast agents, or individual biomedical applications, this review integrates multinuclear NMR and MRI within a single framework spanning materials science and biomedicine. Its novelty lies in linking nucleus selection, sample phase, hardware requirements, and endogenous/exogenous contrast mechanisms as interdependent factors that determine the achievable information content. This cross-domain perspective is intended to provide a unified view of how multinuclear magnetic resonance extends beyond proton-based imaging and spectroscopy. In recent years, advances in methodology and instrumentation have expanded the application of magnetic resonance to complex systems, including functional, porous, composite, and energy-related materials, where classical structural techniques are often insufficient to describe structure–property relationships. In particular, recent progress in ultrafast magic angle spinning (MAS) solid-state NMR (ssNMR) has improved sensitivity, resolution, and acquisition efficiency, thereby expanding the range of complex material systems and dynamic processes accessible to ssNMR [1].

In the case of solid materials, not only is MAS crucial but also specialized high-resolution techniques that address limitations arising, among others, from the presence of quadrupolar nuclei and anisotropic interactions. More broadly, the development of advanced ssNMR hardware and pulse-sequence strategies has expanded the range of systems accessible to high-resolution analysis, particularly in challenging cases involving anisotropic interactions or quadrupolar nuclei [2]. At the same time, ssNMR remains a uniquely useful method for porous and catalytic materials, where it provides information on local structure, host–guest interactions, and atomic-scale dynamics. A good example is its modern application in zeolite chemistry, covering both experimental and methodological aspects [3].

An important aspect of modern magnetic resonance analysis is the shift away from the dominance of a single nucleus (most commonly ^1^H) toward a multinuclear approach. This allows the “observed” nucleus to be selected according to the specific chemical and structural information of interest. From the perspective of both NMR and MRI, the choice of nucleus (e.g., ^1^H, ^13^C, ^31^P, ^23^Na, ^19^F, or others) determines sensitivity, contrast mechanisms, relaxation behavior, and interpretation strategy. In ssNMR, the multinuclear approach is often essential for describing both static and dynamic disorder. It also enables separation of contributions from different material components, as demonstrated, among others, in studies employing parallel use of multiple nuclei in the analysis of complex systems [4].

At the same time, it should be emphasized that NMR is a “phase-universal” technique. It can be applied to gaseous, liquid, and solid samples, with the choice of experimental protocol and instrumentation determined by the nature of the sample and the measurement objective. In the liquid phase, particular importance is given to methods enabling quantitative characterization of molecular transport (e.g., diffusion) with limited sample amounts. This is especially relevant under conditions where the sensitivity of classical experiments is insufficient; examples include sensitivity enhancement strategies in diffusion measurements [5]. In the context of gaseous samples and imaging of transport processes, approaches based on hyperpolarization (HP) are gaining increasing importance. These enable the acquisition of useful image contrast even on clinical scanners typically configured for proton imaging [6].

In the field of MRI, the development of heteronuclear imaging is particularly important, including sodium (^23^Na MRI) and fluorine (^19^F MRI). ^23^Na MRI enables quantitative assessment of sodium concentration and relaxation parameters in selected tissues and organs, as demonstrated in studies of quantitative sodium imaging in humans at 3 T [7]. However, its broader implementation is limited by the intrinsically lower sensitivity of the ^23^Na nucleus compared with ^1^H, rapid relaxation, lower tissue concentration relative to water protons and the resulting need for optimized coils, ultrashort echo-time sequences, and longer acquisition times. At the same time, ^19^F imaging is attractive due to the possibility of signal detection with virtually negligible endogenous background. Nevertheless, ^19^F MRI depends on exogenous fluorinated probes, and its sensitivity is strongly affected by probe concentration, fluorine payload, relaxation properties, and spectral complexity. Therefore, artifact-reduction strategies are required, particularly to address chemical-shift effects and multiple resonance components, as highlighted in recent methodological studies on ^19^F MRI [8]. More broadly, ^19^F-based nanoparticle platforms are being intensively developed as approaches for delivering fluorinated biomaterials and imaging tracers [9].

A common conceptual framework for both NMR and MRI is the distinction between endogenous and exogenous sources of signal. In MRI, an endogenous signal arises from naturally occurring components (e.g., water protons, sodium ions), whereas an exogenous signal appears after the introduction of compounds acting as contrast agents or tracers. The chemistry of contrast agents remains one of the key areas of MRI development. It includes both classical paramagnetic approaches and alternative contrast design strategies. For example, reviews on Fe(III) complexes highlight their potential as contrast agents, emphasizing issues of stability and relaxivity, while reviews on chemical exchange saturation transfer (CEST) MRI systematize a broad range of diamagnetic contrast agents and their contrast-generation mechanisms [10,11]. In parallel, material platforms (e.g., metal–organic frameworks (MOFs)) designed for MR imaging applications are also being developed. This demonstrates that a “material” can simultaneously serve as a functional carrier and as an element generating or modulating contrast [12].

**Figure 1:**
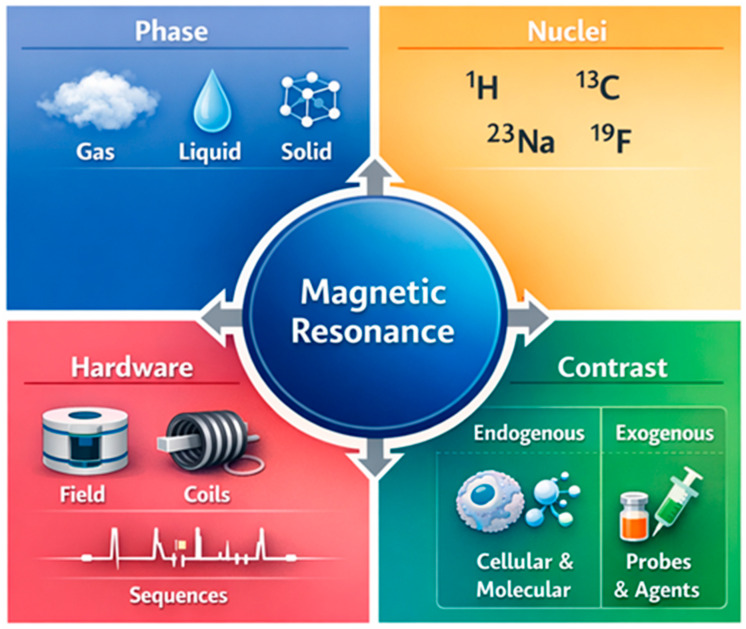
Unified framework of magnetic resonance (MR). The capabilities of NMR and MRI can be understood as the result of four interdependent dimensions: (i) sample phase (gas, liquid, solid), (ii) choice of nucleus (e.g., ^1^H, ^13^C, ^23^Na, ^19^F), (iii) hardware and experimental parameters (magnetic field strength, radiofrequency (RF) coils, pulse sequences), and (iv) contrast mechanisms (endogenous and exogenous). This framework provides a systematic basis for integrating spectroscopic and imaging approaches across chemical, material, and biomedical applications.

In this work, we adopt a perspective in which the capabilities and limitations of NMR/MRI techniques arise from four coupled axes (Figure 1): (i) the sample phase (gas/liquid/solid), (ii) nucleus selection (multinuclear approach), (iii) instrumental solutions (magnetic field strength, probes/coils, gradients, pulse sequences), and (iv) sources of contrast and signal (endogenous vs. exogenous).

## 2. Fundamentals of Magnetic Resonance Relevant to NMR and MR

### 2.1. Nuclear Spin, Resonance Frequency, and Sensitivity

Both NMR spectroscopy and MRI rely on the resonance of nuclei with non-zero spin in an external magnetic field. The resonance frequency is governed by the Larmor relationship and depends on both the magnetic field strength and the gyromagnetic ratio of the observed nucleus.

To provide a concise quantitative comparison, Table 1 summarizes approximate Larmor frequencies of selected MR-relevant nuclei at 3 T. The values were calculated according to Equation (1):

*f* = (*γ*/2π)*B*_0_*,* (1)

where *f* is the resonance frequency, *γ*/2π is the gyromagnetic ratio expressed in MHz/T, and *B*_0_ is the magnetic field strength. The comparison shows that, although ^1^H and ^19^F resonate at relatively high frequencies, nuclei such as ^31^P, ^23^Na, and ^13^C resonate at substantially lower frequencies under the same field conditions.

From a practical perspective, this means that nuclei with high γ (e.g., ^1^H, ^19^F) are easier to excite and detect. In contrast, nuclei with low γ or low natural abundance (e.g., ^13^C, ^31^P, ^23^Na, ^43^Ca, ^103^Rh) impose significantly greater instrumental and methodological demands [12,13,14,15].

The sensitivity of the resonance signal is a multifactorial parameter. It depends not only on γ and magnetic field strength, but also on thermal polarization, linewidth, relaxation times, and RF detection efficiency. In practice, limited sensitivity remains one of the main bottlenecks in both NMR and MRI. This is particularly relevant for complex materials, small sample volumes, or “exotic” nuclei. In recent years, various strategies have been intensively developed to overcome these limitations, including different forms of hyperpolarization. Examples include optically enhanced ssNMR techniques, such as photochemically induced dynamic nuclear polarization (photo-CIDNP), which enable significant enhancement of proton signals in solids using light as an external stimulus [13]. In turn, dissolution-dynamic nuclear polarization (^13^C d-DNP) opens new possibilities for observing metabolic processes in vivo. This demonstrates that sensitivity enhancement is becoming a shared challenge and objective across the entire field of magnetic resonance [16].

### 2.2. Relaxation Mechanisms (T1, T2) in Different Phases of Matter

Nuclear relaxation—described by the time constants T1 (spin–lattice) and T2 (spin–spin)—is a key interpretative element in both NMR and MRI. Relaxation times determine signal intensity, spectral linewidth, and image contrast. Their values are strongly dependent on molecular dynamics and the local nuclear environment. For this reason, relaxation is not merely a technical parameter. It is a carrier of information about structure, mobility, and interactions in the studied system [17,18].

The nature of relaxation mechanisms changes significantly with the phase of matter. In liquids, fluctuations of dipolar interactions modulated by rapid isotropic motion dominate. In solids, restricted dynamics, dipolar coupling networks, and quadrupolar interactions play a key role. In the context of ordered materials, such as lipid membranes or biological structures rich in bound protons, microscopic relaxation models have been proposed. These models account for lateral molecular diffusion and modulation of dipolar interactions, significantly improving the interpretation of T1 and T2 in both MRI and ssNMR [18].

Relaxation also forms the basis of contrast in MRI. T1 and T2 values are sensitive to the presence of paramagnetic centers, chemical composition, hydration level, and microstructure. This sensitivity is exploited in both clinical imaging and materials research. In recent years, quantitative relaxometry and accelerated acquisition and reconstruction methods have gained importance. This includes approaches based on deep learning, which enable rapid mapping of relaxation parameters under conditions of limited signal-to-noise ratio [19]. Similar concepts are applied in ssNMR, where relaxation of nuclei such as ^7^Li or ^2^H is used as a probe of ionic dynamics or transport processes in functional materials [20,21].

### 2.3. Chemical Shift and Local Chemical Environment

Chemical shift is the central observable of NMR spectroscopy and the primary source of chemical information. Its value reflects the magnetic shielding of a nucleus by the surrounding electron cloud. Consequently, it encodes the local chemical environment, including bonding type, protonation state, coordination, intermolecular interactions, and the presence of structural defects. In materials analysis, chemical shift acts as a local “structure sensor”. It enables differentiation of nonequivalent atomic sites even in amorphous or highly disordered systems [22,23,24,25,26].

In this context, quantum-chemical calculations, particularly density functional theory (DFT), provide an important complementary tool for NMR interpretation. Calculated shielding tensors and chemical shifts can support signal assignment, validate proposed local structures, and help distinguish between structurally similar environments, especially in solids, amorphous materials, porous systems, and cases where experimental spectra are strongly overlapped. Thus, although the present review focuses primarily on experimental NMR and MRI, DFT-assisted analysis represents an important bridge between measured NMR parameters and atomistic structural models [23,24,25].

At the same time, interpretation of chemical shifts requires caution, as they are sensitive to experimental conditions such as pH, temperature, and matrix composition. Systematic studies of chemical shift variations in common metabolites show that even small environmental changes can lead to significant shifts and line broadening. This has implications for both spectroscopy and quantitative applications [26]. In complex systems, such as glycans or mixtures with highly degenerate spectra, chemical and synthetic strategies are being developed to “disentangle” chemical shift degeneracy. This increases the utility of NMR as a structural tool [27].

A special case involves systems containing paramagnetic centers, where chemical shifts can be strongly affected by contact and pseudocontact interactions. Although these effects often complicate spectra, modern treatments of paramagnetism in NMR show that they can provide unique information about electronic structure and the geometry of the metal environment. This is particularly relevant in materials chemistry and catalysis [28].

### 2.4. Multinuclear Magnetic Resonance—Advantages and Limitations

The multinuclear approach is one of the key directions in the development of both NMR and MRI. The choice of observed nucleus determines the type of information obtained. Protons provide high sensitivity, whereas nuclei such as ^13^C, ^31^P, ^23^Na, ^19^F, or ^7^Li enable selective investigation of chemical composition, ion transport, metabolism, or non-proton local environments [29]. In MRI, heteronuclear imaging opens the possibility of background-free contrast (e.g., ^19^F) or quantitative assessment of concentrations and relaxation parameters (e.g., ^23^Na MRI).

However, the advantages of the multinuclear approach are accompanied by significant limitations. The low natural abundance and low γ of many nuclei result in weak signals, long acquisition times, and increased hardware requirements. In ssNMR, quadrupolar nuclei are particularly challenging, as strong interactions lead to substantial line broadening. In response, strategies such as indirect detection and multidimensional correlation experiments have been developed. These approaches transfer information from a difficult nucleus to one that is easier to detect, as demonstrated for systems involving ^19^F, ^15^N, or metal nuclei [30,31,32].

In MRI, multinuclear approaches require trade-offs between sensitivity, acquisition time, and RF power constraints. An example is interleaved protocols combining ^23^Na MRI and ^31^P MR spectroscopic imaging (MRSI) within a single experiment. These demonstrate that integration of multiple nuclei is feasible, but requires precise optimization of instrumentation and pulse sequences [33]. At the same time, advanced signal processing, including denoising methods, is becoming increasingly important as an integral component of improving effective sensitivity in multinuclear MR experiments [34].

## 3. NMR Spectroscopy Depending on the Sample Phase

**Figure 2:**
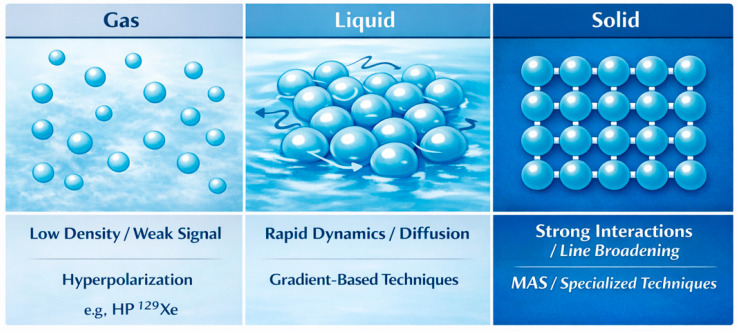
Magnetic resonance across different phases of matter. The characteristics of MR signals depend strongly on the physical state of the sample. In gases, low spin density and rapid diffusion lead to weak signals, often requiring hyperpolarization (e.g., HP ^129^Xe). In liquids, fast molecular motion averages anisotropic interactions, enabling high-resolution spectroscopy and diffusion measurements. In solids, strong dipolar and anisotropic interactions dominate, leading to line broadening and necessitating specialized techniques such as MAS.

The properties of the NMR signal are strongly dependent on the phase of matter, as molecular dynamics and the nature of spin interactions differ fundamentally between gases, liquids, and solids. These differences determine both experimental strategies and the quality of the information obtained. These relationships are illustrated schematically in Figure 2.

### 3.1. NMR of Gaseous Samples

#### 3.1.1. Signal Characteristics

Gas-phase NMR represents a special case of magnetic resonance spectroscopy in which the observability and quality of spectra are determined by: low spin density, rapid translational diffusion, and sensitivity to magnetic-field inhomogeneities. Consequently, gas-phase experiments generally require high field stability, careful shimming, and optimized sample geometry.

The gas phase is also particularly useful for shielding metrology and computational validation, because reduced intermolecular interactions make chemical shifts and coupling constants closer to isolated-molecule values. Reviews devoted to magnetic shielding measurements and to the determination of optimal shielding and spin–spin coupling parameters in fluorinated compounds emphasize that gas-phase data can serve as a reference for consistent chemical shift scales and for testing quantum-chemical methods [35,36].

In practical applications of “gas MR”, the most significant breakthrough in sensitivity and accessibility has been achieved through hyperpolarization of noble gases (primarily ^129^Xe, historically also ^3^He). 

This enables otherwise weak gas signals to be detected and makes ^129^Xe a sensitive probe of local environments because of its broad chemical-shift range and solubility in different phases [37,38]. Hyperpolarized CEST further extends this concept by exploiting saturation transfer and chemical exchange to detect small xenon populations bound to carriers such as porous hosts [39].

#### 3.1.2. Applications

(A) Gas phase as a metrological reference and source of fundamental parameters

As indicated by reviews in the field of magnetic shielding, gas-phase NMR enables separation of intermolecular contributions and provides data closer to intrinsic molecular properties. Such data support chemical-shift/shielding databases and validation of quantum-chemical calculations [35,36].

(B) Investigation of gas transport and diffusion (porous materials, adsorption, separation)

In materials science, a key application of gas-phase NMR is the study of sorption and transport in porous materials (zeolites, MOFs, carbon sorbents, porous silicas). MR methods provide simultaneous insight into: (i) speciation and binding modes of adsorbates, (ii) molecular dynamics and mobility within pores, and (iii) heterogeneity of adsorption environments.

A review on CO_2_ capture studies using NMR summarizes practical aspects of such experiments. It highlights, among others, the role of pulsed-field gradient (PFG) NMR in diffusion measurements and the limitations of simple one-dimensional ^13^C approaches in unambiguously determining CO_2_ binding mechanisms [40]. In the same context, a review on the structure and speciation of CO_2_ in silica-based adsorbents shows that combining ssNMR with computational modeling enables disentangling parallel adsorption pathways and chemical transformations (e.g., carbonate/bicarbonate species) in heterogeneous materials [41].

Additionally, studies presenting “adsorption-assisted” approaches demonstrate that NMR can be used directly to obtain adsorption isotherms. This provides an attractive alternative or complement to classical volumetric methods, particularly when simultaneous information about the chemical state of the adsorbate is required [42].

An important related area is catalysis. Reviews on mass transport in porous catalysts emphasize that gas-phase diffusion plays a critical role in macro- and microkinetics and in the effective utilization of active sites within pores [43].

(C) Hyperpolarized ^129^Xe as a probe of the gas phase and gas–solid/liquid interfaces

From a materials perspective, its key advantages are hyperpolarization-enhanced sensitivity and strong environmental dependence of the chemical shift.

Reviews of preclinical HP ^129^Xe MRI demonstrate how diffusion and gas exchange can be probed on short spatial scales, and how spectral signatures related to gas distribution and transfer into the dissolved phase are interpreted [37]. In parallel, reviews addressing the integration of clinical technologies into hyperpolarized ^129^Xe MR highlight the methodological maturity and transferability of this approach (coils, sequences, reconstruction). This is also relevant for materials research under conditions of limited sensitivity [38].

Finally, HyperCEST represents a strictly “molecular–materials” platform, in which carriers (e.g., porous hosts) become an integral part of the contrast-generation mechanism [39].

#### 3.1.3. Experimental Limitations

(A) Low sensitivity and Signal-to-Noise Ratio (SNR) requirements

In gases, the limitation arises from the low number of spins within the sample volume and often shorter effective observation times (due to diffusion and dephasing in inhomogeneities). This is commonly addressed by increased pressure, larger or high-filling-factor coils, efficient pulse sequences, and hyperpolarization where applicable [37,38].

(B) Sensitivity to gradients, diffusion, and off-resonance artifacts

Rapid gas diffusion makes even small B_0_ inhomogeneities and gradients (intentional or not) a source of signal decay, phase errors, and reconstruction artifacts. In HP ^129^Xe imaging, contamination from gas-phase signals and off-resonance components can interfere with quantification of gas exchange. It has been shown that appropriate processing and referencing strategies can reduce these artifacts without major modifications to pulse sequences [43,44].

Therefore, careful control of frequency drift, excitation selectivity, and multicomponent signal decomposition is essential [37,44].

(C) Geometric constraints and experimental conditions (flow, pressure, sample compatibility)

Gas-phase experiments often require high-pressure NMR cells, flow control, temperature stabilization, and chemically compatible materials (to avoid wall adsorption or permeation). High-pressure experiments with gases (e.g., p-H_2_) using dedicated cells and probes illustrate the importance of hardware solutions in extending accessible experimental conditions [45].

(D) Specificity for porous materials: heterogeneity and interpretation

In porous materials, the observed signal is typically a sum of contributions from multiple adsorption environments and dynamic states. Reliable interpretation therefore requires multidimensional experiments, pressure- or temperature-dependent measurements, PFG methods, and integration with modeling to avoid overinterpretation of simple observables such as single chemical shifts [40,41].

Moreover, when NMR is used for direct reconstruction of adsorption isotherms, validation against classical methods and strict control of equilibrium conditions are necessary [42].

### 3.2. NMR of Liquid Samples

NMR in the liquid phase remains a reference method in chemistry and biophysics. It enables non-destructive measurements that simultaneously provide structural, quantitative, and dynamic information under conditions close to the natural environment of the sample. In practice, “liquid samples” include not only classical solutions of small molecules, but also more complex systems such as reaction mixtures or environmental matrices. A key aspect is the appropriate selection of one-dimensional and multidimensional experiments, as well as acquisition and data-processing strategies. These allow maximization of information content while maintaining an adequate signal-to-noise ratio and reasonable acquisition times [46,47,48].

Classical NMR serves as a benchmark for spectroscopic interpretation, providing high resolution and reliable structural assignments. Standard one-dimensional (1D) (^1^H, ^13^C) and two-dimensional (2D) correlation experiments enable structural identification, kinetic analysis, and quantitative determination of sample composition. In laboratory practice, classical pulse sequences are often treated as the “gold standard” against which new methodological approaches are evaluated. Methodological reviews emphasize the importance of NMR as a tool for mechanistic analysis and validation of structural assignments, particularly in complex chemical systems [46].

At the same time, advances in instrumentation, including benchtop systems, are expanding the possibilities for rapid and non-destructive sample screening. However, high-field spectrometers remain essential for achieving the highest spectral resolution [49,50].

One of the key advantages of solution NMR is the ability to directly probe molecular dynamics. Relaxation parameters, diffusion measurements, and observations of chemical exchange provide information on rotational and translational motions, as well as intermolecular interactions. Ultrafast approaches to relaxation and diffusion measurements significantly reduce acquisition times, enabling the study of non-stationary and rapidly evolving systems [51].

NMR relaxometry, including measurements over a wide frequency range, provides a sensitive tool for studying the dynamics of complex liquids such as ionic liquids or glass-forming systems. Analysis of relaxation spectra enables correlation of molecular mobility with structural interactions [52,53]. Additionally, magnetic field manipulation, such as in field-cycling experiments, allows probing subtle aspects of enzymatic dynamics and ligand-binding processes [54].

Spectral resolution remains one of the most important parameters in liquid-state NMR. High magnetic field homogeneity and rapid averaging of anisotropic interactions result in narrow spectral lines. However, signal overlap remains a challenge in complex mixtures. Solutions include multidimensional experiments and advanced acquisition strategies. Ultrafast 2D NMR enables acquisition of full correlation spectra in a single scan, significantly reducing measurement time while preserving structural information [47,55]. Methods for mixture analysis emphasize the need for an optimal balance between sensitivity, resolution, and experimental time [48].

Alternative approaches include “pure shift” techniques, which suppress homonuclear scalar couplings and reduce multiplet structure. This leads to significantly improved effective resolution and facilitates analysis of biological and pharmaceutical samples [56,57,58]. In addition, machine learning tools are increasingly used for data reconstruction, denoising, and automated spectral assignment, improving both effective resolution and reliability of analysis [59].

#### 3.2.1. Signal Characteristics and Classical NMR as a Reference

NMR in the liquid phase remains one of the most important spectroscopic methods in chemistry, biophysics, and materials science, as it enables simultaneous acquisition of structural, quantitative, and dynamic information under conditions close to the natural environment of the sample. In practice, the term “liquid samples” encompasses a wide range of systems, from simple solutions of small molecules to more complex systems such as reaction mixtures, biological systems, or environmental matrices.

Under these conditions, rapid rotational and translational motion leads to effective averaging of anisotropic interactions, resulting in narrow spectral lines and high signal resolution. As a result, solution-state NMR serves as a reference method for structural interpretation and signal assignment in more complex systems [46].

Classical one-dimensional experiments, such as ^1^H and ^13^C NMR spectra, combined with 2D correlation experiments, enable structural identification, mechanistic analysis of reactions, and quantitative determination of mixture composition. In many laboratories, the set of standard pulse sequences is treated as the “gold standard” of chemical spectroscopy. New methodological developments are typically evaluated against this benchmark. Reviews on mechanistic analysis using NMR emphasize that the method remains one of the most universal tools for studying reaction pathways and identifying intermediates in real time [46].

At the same time, advances in instrumentation, including both high-field spectrometers and compact benchtop systems, expand the applicability of NMR in rapid screening and routine analysis. Nevertheless, the highest spectral resolution is still achieved with high-field instruments [49,50].

#### 3.2.2. Molecular Dynamics in Solution

One of the most important advantages of solution-state NMR is the ability to directly investigate molecular dynamics across a wide range of timescales. Relaxation parameters, diffusion measurements, and observations of chemical exchange processes provide insight into rotational and translational motions of molecules, as well as intermolecular interactions within the system. As a result, NMR serves as a sensitive tool for studying the dynamics of both simple and complex liquids, including biological systems, ionic liquids, and glass-forming materials [51,52,53].

In recent years, ultrafast approaches to relaxation and diffusion measurements have gained particular importance, enabling significant reduction in data acquisition times. These methods allow observation of processes occurring in non-stationary systems, such as chemical reactions or transport processes, in real time while maintaining high dynamic resolution [51,55].

In studies of biological and enzymatic systems, additional analytical capabilities are provided by field-cycling NMR experiments. By manipulating the magnetic field strength, these experiments reveal subtle aspects of ligand–protein interactions and catalytic processes [54].

#### 3.2.3. Spectral Resolution and New Acquisition Strategies

High spectral resolution is one of the key advantages of liquid-state NMR. Due to magnetic field homogeneity and averaging of anisotropic interactions, very narrow resonance lines can be obtained. However, in complex chemical mixtures, signal overlap remains a significant challenge. To address these limitations, new acquisition and data analysis strategies are being developed, aiming to improve effective spectral resolution while reducing experimental time.

One of the most important developments is ultrafast 2D NMR, which enables acquisition of full correlation spectra in a single scan. This approach significantly reduces measurement time while preserving structural information, which is particularly important in the analysis of reaction mixtures and dynamic systems [47,55].

In mixture analysis, emphasis is placed on achieving an optimal balance between sensitivity, resolution, and experimental time, as each of these parameters affects the reliability of spectral interpretation [48].

An alternative strategy for improving resolution is the use of “pure shift” techniques, which reduce multiplet structures arising from homonuclear couplings. As a result, spectra with significantly higher effective resolution are obtained, facilitating analysis of biological, metabolomic, and pharmaceutical samples [56,57,58].

In parallel, machine learning-based tools are becoming increasingly important. They support data reconstruction, denoising, and automated signal assignment. Integration of computational methods with classical NMR analysis enhances both effective resolution and reliability, particularly for large datasets and complex mixtures [59].

### 3.3. Solid-State NMR

Solid-state NMR spectroscopy is one of the most important methods for studying materials whose structure or properties prevent analysis under solution conditions. This applies particularly to amorphous materials, polymers, heterogeneous catalysts, solid electrolytes, porous materials, nanostructures, and pharmaceutical solids, including active pharmaceutical ingredients (APIs) and their solid dosage forms. In such systems, the technique provides direct insight into the local atomic environment and molecular dynamics without the need to dissolve or chemically modify the sample. This is particularly important in pharmaceutical analysis, where dissolution may erase information on polymorphism, salt formation, crystallinity, hydration state, and static or dynamic disorder. Multinuclear ssNMR, including ^1^H, ^13^C, and heteronuclear detection, can therefore be used to characterize molecular packing, local disorder, and phase heterogeneity in solid APIs, as demonstrated for pharmaceutical hydrochlorides [4]. As a result, ssNMR delivers information on chemical structure, intermolecular interactions, and molecular mobility at the atomic scale, making it particularly valuable in studies of functional, catalytic, and pharmaceutical materials [1,4,60,61,62].

However, NMR measurements in solids are associated with several challenges that distinguish this method from solution spectroscopy. In liquids, rapid molecular motion averages many anisotropic interactions, resulting in narrow and well-resolved spectral lines. In solids, this averaging mechanism is absent, and observed signals are often significantly broadened. Line shape and width are primarily influenced by chemical shift anisotropy, dipolar couplings, and—especially for quadrupolar nuclei—interactions with electric field gradients. Consequently, obtaining high-resolution spectra requires appropriate experimental strategies and advanced instrumentation.

In some cases, additional complications arise from the presence of paramagnetic centers, which can lead to very large chemical shifts and shortened relaxation times, making structural interpretation more difficult [63]. Similar issues occur in experiments conducted at cryogenic temperatures, where increased stability of intermediate states is often accompanied by additional line broadening [64].

Dipolar interactions between nuclear spins are of particular importance in ssNMR. In solids, they are not fully averaged by molecular motion and therefore strongly influence linewidth and spectral complexity. At the same time, these interactions carry important structural information, as their magnitude depends directly on internuclear distances and orientations. Analysis of dipolar couplings thus enables inference of local geometry and molecular dynamics. In nanoporous systems or materials with restricted molecular mobility, partial averaging of dipolar interactions may reflect molecular motion within pores and interactions with material surfaces [65].

In practice, numerous experimental methods have been developed to control dipolar interactions. One commonly used strategy is selective “recoupling” of these interactions under conditions where they have been partially averaged. Techniques such as rotational-echo double resonance (REDOR) enable measurement of distances between selected nuclei by controlled reintroduction of dipolar couplings during the experiment. This method has been widely applied in studies of biomolecules and functional materials, providing information on interatomic distances and structural organization [66].

More advanced polarization transfer schemes and pulse sequences are also being developed, improving sensitivity and enabling more efficient use of heteronuclear correlations in multidimensional experiments [67].

The most important technique enabling high resolution in solid-state NMR is MAS. Rotation of the sample at high speed around an axis oriented at 54.74° relative to the magnetic field averages many anisotropic interactions responsible for line broadening. As a result, ssNMR spectra can achieve resolution comparable to that observed in solution NMR, greatly facilitating signal assignment and structural analysis [61].

Recent technological advances have led to the development of fast and ultrafast MAS experiments, enabling efficient proton detection in solids and the implementation of complex multidimensional experiments [68,69].

Despite the effectiveness of MAS, dipolar interactions are not completely eliminated, particularly in systems with high proton density. Even at very high spinning speeds, residual dipolar linewidth may remain, arising from the properties of the dipolar Hamiltonian and strong spin coupling. This phenomenon defines the practical limits of proton spectral resolution in ssNMR and motivates continued development of approaches such as sample deuteration, selective isotopic labeling, and experiments at even higher MAS frequencies [70].

As a result, modern solid-state NMR constitutes a comprehensive set of methods for studying the structure and dynamics of materials at the atomic level. The combination of advanced instrumentation, control of spin interactions, and sample preparation strategies enables analysis of increasingly complex chemical and biological systems. Consequently, ssNMR remains one of the key techniques for material characterization, particularly where other structural methods are insufficient [60,61,62,63,64,65,66,67,68,69,70].

The characteristics of the MR signal depend strongly on the physical state of the system, as molecular motion and intermolecular interactions differ significantly between gases, liquids, and solids. These differences affect both the choice of measurement methods and the quality of the obtained signal. The key features and limitations associated with different states of matter are summarized in Table 2.

## 4. Multinuclear Magnetic Resonance in Hard and Soft Materials

**Figure 3:**
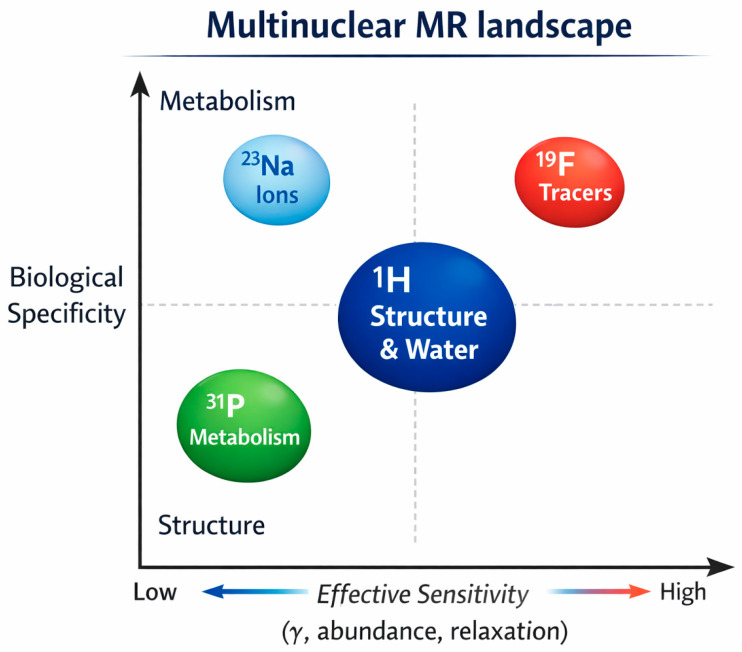
Multinuclear MR landscape. Different nuclei provide complementary information in magnetic resonance, spanning structural, metabolic, and molecular domains. Proton (^1^H) MRI offers high sensitivity and anatomical detail, while nuclei such as ^23^Na and ^31^P provide insights into ion homeostasis and energy metabolism. ^19^F enables highly specific molecular imaging using exogenous tracers. The positioning reflects a trade-off between effective sensitivity (determined by gyromagnetic ratio, natural abundance, and relaxation properties) and biological specificity.

Magnetic resonance extends beyond conventional proton imaging, enabling access to a wide range of nuclei with different physical properties and biological relevance. These nuclei differ not only in sensitivity, but also in the type of information they provide, ranging from structural imaging to metabolic and molecular insights. This multidimensional landscape is schematically illustrated in Figure 3.

### 4.1. Proton (^1^H) NMR

Proton magnetic resonance remains the most widely used form of NMR in studies of hard and soft materials. This is primarily due to the very high sensitivity of the ^1^H nucleus, its ubiquitous presence in organic materials, and the direct relationship between the proton signal and local segmental mobility, degree of hydration, and microstructural heterogeneity. In practice, this means that ^1^H NMR can play a dual role. On the one hand, it provides information on the dynamics of polymer chains themselves. On the other, it enables tracking of the state and distribution of water, which in many soft matter systems becomes a sensitive marker of spatial organization [71,72,73].

One of the most important applications of proton NMR in solid and semi-solid materials is the assessment of segmental mobility. Relaxation parameters, FID decay shape, linewidth, and multiple-quantum experiments are strongly dependent on the amplitude and timescale of molecular motions. Therefore, they enable differentiation between rigid, intermediate, and highly mobile domains. For example, in studies of poly(vinyl butyral), it has been shown that combining time-domain ^1^H NMR techniques with relaxation measurements allows separate analysis of backbone and side-chain motions. This enables a more precise description of local polymer dynamics [71].

Similarly, in elastomers, proton relaxometry and analysis of residual dipolar couplings enable correlation of the NMR response with crosslinking density, topological constraints, and the relative contributions of rigid and mobile fractions [72,73]. The importance of this approach is further confirmed by RheoNMR studies of hydrogels, where changes in the ^1^H signal were monitored alongside the evolution of mechanical properties. It was demonstrated that the increase in material modulus is directly correlated with progressive restriction of polymer segment mobility [74].

In solid materials, proton NMR is particularly useful when classical high-resolution spectral analysis is hindered by strong dipolar interactions and local heterogeneity. Under such conditions, information is derived not only from signal position but primarily from signal decay and relaxation behavior. In partially disentangled ultrahigh-molecular-weight polyethylene (UHMWPE), low-field ^1^H ssNMR enabled observation of changes in chain segment mobility near the melting temperature and demonstrated the influence of entanglement density on amorphous-phase dynamics and chain reorganization [75].

Similarly, analysis of large datasets of static ^1^H ssNMR spectra for various polymer materials has shown that the proton signal can be used to classify mobile, rigid, and intermediate domains. This allows characterization of domain structure without the need for labeling or external probes [76]. In this sense, ^1^H NMR provides direct insight into the “intrinsic” dynamics of the studied system.

A second key area of application of proton NMR is the analysis of water as a structural marker. In soft materials, water is not merely part of the measurement environment, but an integral component of material organization. Protons of water molecules respond to restricted motion, interactions with polar groups, confinement in pores, and exchange with polymer protons. Therefore, T1 and T2 relaxation times and their distributions can be interpreted as indirect signatures of microstructure and hydration state [77,78].

In alginate matrices, low-field ^1^H relaxometry enabled simultaneous monitoring of hydration and polymer mobilization. It was shown that an increase in total signal intensity and changes in relaxation parameters reflect structural reorganization during swelling [77]. A similar approach was applied to polyethyleneimine hydrogels, where proton NMR revealed changes in water mobility and confinement after ionic crosslinking. These changes were directly related to network density and transport properties of the material [78].

Particularly useful in this context is the distinction between free, bound, and immobilized water fractions. In practice, T2 distributions often reveal multiple proton populations. These can be assigned to strongly bound water at macromolecular surfaces, partially restricted water, and more mobile water. This approach has been applied both in polymer materials design, where complex T2 relaxation curves were decomposed into bound and free components to correlate with material properties [79], and in studies of hydrogels and protein gels, where water dynamics reflected network density, porosity, and structural organization [80,81].

For example, in collagen gels, relaxation data have been interpreted in terms of water–macromolecule interactions and diffusion in confined environments. This reinforces the role of water as a sensitive indicator of material state [81].

In porous and composite systems, the proton signal originating from water also serves as an indicator of accessibility and nature of internal spaces. Even when the solid framework produces a weak or broad signal, protons of the liquid phase within pores can provide information about pore size, surface affinity, and wettability. In wet carbon materials, ^1^H relaxometry enabled differentiation between surface-bound and more mobile water populations, allowing indirect characterization of heterogeneous porosity [82].

A similar interpretative framework applies to soft polymer membranes, where water mobility is strongly coupled to local ordering of hydrophilic domains and to transport properties of the system [83].

The ubiquity of protons means that in many applications, ^1^H NMR relies on the endogenous signal. This signal originates directly from the material itself or from naturally present water, without the need for exogenous labels. In polymer materials, endogenous protons include those of the backbone, side groups, and sorbed, capillary, or bound water molecules. As a result, proton NMR can be considered a non-destructive and minimally invasive technique. This is particularly valuable when the introduction of external probes could disturb system equilibrium or alter physicochemical properties [71,76,77].

In practice, this means that contrast in ^1^H NMR arises naturally from differences in mobility, hydration, diffusion restriction, and local chemical environment.

From the perspective of hard and soft materials, proton NMR thus acts as a bridge between classical structural analysis and functional characterization of material dynamics. It enables simultaneous description of polymer segment mobility, water state, and domain heterogeneity, with the observed signal being largely endogenous. This feature makes ^1^H NMR particularly useful in studies of hydrogels, elastomers, membranes, porous materials, and other soft matter systems, where macroscopic properties are directly governed by local molecular dynamics and the organization of the aqueous phase [72,73,74,75,76,77,78,79,80,81,82,83].

### 4.2. Carbon (^13^C) NMR

In studies of hard and soft materials, ^13^C NMR spectroscopy plays a particularly important role when the goal is reconstruction of the chemical backbone and assessment of structural order. In contrast to proton NMR, which is highly sensitive to segmental mobility and hydration state, the ^13^C signal is more strongly related to chain topology, functional group type, degree of crosslinking, and the local environment of carbon atoms.

For this reason, ^13^C NMR—especially in the solid-state variant with magic-angle spinning and cross-polarization techniques—is one of the primary methods for describing the structure of polymers, biopolymers, and porous materials. This is particularly relevant when classical solution analysis is not possible or would lead to loss of native structural information [84,85].

A key advantage of ^13^C NMR is the ability to distinguish different types of carbon atoms belonging to the backbone, side groups, aromatic, carbonyl, or aliphatic fragments. This allows not only confirmation of chemical composition, but also reconstruction of connectivity and detection of structural changes arising during synthesis, processing, or degradation [86].

For example, in poly(glycerol citrate) polyester, detailed ^13^C NMR analysis enabled identification of dominant ester linkages and evaluation of how synthesis conditions affect macromolecular architecture [87]. Similarly, in highly branched tannin-based wood adhesives, ^13^C NMR revealed the dominant crosslinking mechanism, primarily involving substitution of phenolic hydroxyl groups by amine functionalities. This allowed direct correlation between chemical structure and material properties [88].

In more complex materials, especially natural and renewable systems, ^13^C ssNMR is particularly valuable because it enables analysis in a near-native state without dissolution or extensive chemical treatment. This applies especially to lignocellulosic materials, polysaccharides, and other heterogeneous systems, where backbone structure is closely linked to function.

Reviews on cellulose-based and bioenergy materials emphasize that ^13^C ssNMR allows identification of cell wall components, differentiation of carbon environments, and monitoring of structural reorganization at the molecular level without sample destruction [84,85]. This is well illustrated by studies of plant cellulose, where two-dimensional ^13^C ssNMR enabled differentiation of multiple glucose environments and provided a more detailed picture of microfibril organization [89].

Another key application is the assessment of structural order. In polymer materials, this includes the fraction of crystalline and amorphous phases, chain regularity, local domain organization, and microstructural heterogeneity. ^13^C NMR is particularly useful here because chemical shift, linewidth, and relative signal intensities reflect local conformational and spatial order.

In cellulose materials, ssNMR has long been used to distinguish more ordered and less ordered regions. Recent studies further demonstrate that subtle differences in ^13^C environments can be used to describe microfibril organization and relationships between surface and core regions [84,89].

The importance of ^13^C NMR in assessing order is also evident in polysaccharides and biological materials. Cross-polarization magic angle spinning (CP-MAS) ^13^C NMR analysis of chitosan oligomers has shown that the technique can detect structural changes resulting from different hydrolysis mechanisms, reflecting alterations in backbone organization [90].

In porous organic polymers with azo linkages, ^13^C CP-MAS NMR has been used as a key method to confirm network structure and material homogeneity, which is essential for interpreting sorption properties [91]. In such systems, the ^13^C signal allows differentiation between well-defined network structures and materials with higher defect content or disrupted local organization.

Importantly, ^13^C ssNMR enables analysis of materials with partial or local order. These are systems that are not fully crystalline but contain domains with varying degrees of organization. This includes many hydrogels, biopolymers, porous materials, and partially crosslinked systems.

In such cases, ^13^C NMR provides information complementary to diffraction methods. Instead of requiring long-range order, it probes the local atomic environment. This makes it particularly valuable for analyzing amorphous and semi-ordered materials, where it can detect differences in local backbone organization even when diffraction techniques provide limited information [85,92].

In a broader perspective, ^13^C NMR in hard and soft materials is primarily a structural tool. It enables identification of fundamental building units, determination of their connectivity, and assessment of the degree of order versus amorphousness, heterogeneity, or local reorganization.

Thus, it naturally complements proton NMR: while ^1^H NMR is more sensitive to mobility and proton phase behavior, ^13^C NMR provides more direct insight into the carbon framework, i.e., the structural basis of material properties [84,85,86,87,88,89,90,91,92].

### 4.3. Phosphorus (^31^P) NMR

^31^P NMR spectroscopy occupies a special place among multinuclear techniques, as it combines relatively good sensitivity with high chemical selectivity toward phosphate, phosphonate, and phosphite groups. This makes it particularly useful in studies of functional materials in which phosphorus atoms form part of the chemical backbone, act as crosslinking groups, active centers, or tracers.

In practice, ^31^P NMR is used both for identifying phosphorus chemical species and assessing the degree of material modification, as well as for probing the local environment, segmental dynamics, and transformations occurring during synthesis, processing, and application [93,94,95].

In functional materials, polymers and networks containing phosphorus are of particular importance. Their properties—such as hydrophilicity, self-assembly, ionic conductivity, degradability, and functionalization potential—are closely related to the type and environment of phosphorus groups. In such systems, ^31^P NMR allows direct monitoring of P–O and P–C bonds.

For example, in RNA-inspired dynamic covalent networks, the use of anionic phosphate diesters as the sole dynamic crosslinking unit enabled the formation of reprocessable materials. ^31^P NMR was essential for confirming chemical structure and monitoring phosphorus group behavior [94]. Similarly, in thermoresponsive polyphosphoesters, NMR analysis confirmed macromolecular structure and linked phosphorus segment architecture to self-assembly in aqueous environments [95].

In materials designed for ion storage and transport, ^31^P NMR provides insight not only into chemical structure, but also into the local environment governing ionic mobility. Reviews on solid electrolytes highlight the importance of NMR, including phosphorus nuclei, for studying solvation, mobility, and organization of conductive phases in ion-exchange membranes and composite materials [93].

In such systems, phosphorus groups are often integral to the transport-active phase. Therefore, the observed signal directly reflects local chemistry rather than indirect structural features.

In biomaterials, ^31^P NMR has a dual role. First, it enables analysis of materials containing phosphate-based mineral phases, such as hydroxyapatite, amorphous calcium phosphate, or phosphate glasses. Second, it is highly useful for studying polymer materials modified with phosphorus groups to improve biocompatibility, degradability, or functionality.

For example, in polyvinylpyrrolidone–hydroxyapatite composites, MR techniques were used to characterize materials intended for regenerative applications, demonstrating the value of a multinuclear approach in describing polymer–mineral interfaces [96]. In materials based on amorphous calcium phosphate combined with organic molecules, combined ^13^C and ^31^P ssNMR enabled detailed characterization of mineral phase structure and transformations, which is crucial for biomimetic material design [97].

Similarly, ^31^P MAS NMR was used in highly porous phosphate glasses for controlled copper ion release, allowing analysis of phosphate network organization responsible for degradation and release behavior [98].

Phosphorus-containing polysaccharide and polymer membranes also represent an important class of biomaterials. In such systems, phosphorus groups influence crosslinking, hydrophilicity, and barrier properties. In starch-based membranes crosslinked with hypophosphite/chitosan systems, combined ^13^C and ^31^P CP-MAS NMR confirmed network structure and chemical changes associated with phosphorylation [99].

A rapidly developing area involves phosphorus-containing polymers as probes and carriers for ^31^P MRI/MRS. In these systems, phosphorus acts not only as a structural element, but also as a source of imaging signal. This is particularly attractive because the ^31^P signal can be detected with very low biological background.

Studies have shown that appropriately designed phosphorus-containing polymers can serve as sensitive and biocompatible ^31^P MR probes [100]. Biodegradable polyphosphoester micelles can function simultaneously as drug carriers and imaging agents, enabling in vivo tracking without metal-based contrast agents [101]. Gradient polyphosphoester copolymers further demonstrate the possibility of designing materials with tunable interfacial properties and imaging capability [102].

In biological membranes and model phospholipid systems, ^31^P NMR remains a fundamental tool for studying lipid phase organization, bilayer symmetry, membrane curvature, and interactions with peptides or small molecules. Because phosphate groups are integral to phospholipid headgroups, the ^31^P signal directly reflects membrane organization.

For example, studies of antimicrobial peptide interactions with erythrocyte membranes used in situ ^31^P and ^2^H ssNMR to describe peptide-induced lipid reorganization [103]. Similar approaches have been applied to study HIV gp41 interactions with membranes, where ^31^P ssNMR provided insight into membrane curvature and fusion-related changes [104]. Reviews on small-molecule–membrane interactions emphasize that the ^31^P signal provides key information on phase state, orientation, and bilayer perturbation [105].

In summary, ^31^P NMR is particularly useful in functional materials, biomaterials, and membrane studies because the observed nucleus is often directly associated with chemically and functionally critical components. It enables structural validation, monitoring of modifications, and analysis of network reorganization in polymers; characterization of mineral phases in biomaterials; and direct probing of membrane organization.

Furthermore, the development of phosphorus-containing materials opens the way for integration of ^31^P NMR with heteronuclear MR imaging, highlighting its importance in modern materials and biomedical research [93,94,95,96,97,98,99,100,101,102,103,104,105].

### 4.4. Other Important Nuclei (^23^Na, ^19^F)

In addition to ^1^H, ^13^C, and ^31^P, nuclei such as ^23^Na and ^19^F play an important role in materials and biomedical research. Their significance arises from the fact that they provide information not accessible, or poorly visible, in conventional proton imaging. Sodium is directly related to ion transport, osmotic homeostasis, and cellular metabolism, whereas fluorine enables imaging of exogenously labeled systems with virtually zero biological background.

Thus, the choice of nucleus in multinuclear studies is not merely a matter of instrumentation availability, but results from a trade-off between sensitivity, natural abundance, spin properties, presence in the system, and the type of information required [102,103,104].

A primary criterion for nucleus selection is its informational relevance to the research problem. If the goal is to assess endogenous ion distribution and cellular homeostasis, ^23^Na is more appropriate. If the aim is to track an exogenous tracer, nanocarrier, or labeled cells without background interference, ^19^F is preferred [106,107,108,109].

Reviews on X-nuclei imaging emphasize that four main factors determine nucleus selection: physical properties, concentration in the system, availability of hardware and sequences, and biological or material relevance of the signal [106,108]. In this sense, ^23^Na and ^19^F represent two complementary paradigms: endogenous versus exogenous signal.

^23^Na is particularly attractive because sodium plays a fundamental physiological and material role. Its local concentration is a sensitive indicator of metabolic changes, cellular integrity, ion transport, and hydration state. However, as a quadrupolar nucleus (spin 3/2), it exhibits more complex relaxation behavior, shorter T2 times, and greater experimental challenges than spin-1/2 nuclei [107,110].

This results in a lower signal-to-noise ratio, the need for fast sequences, ultrashort echo times, and often high magnetic fields. At the same time, these properties make ^23^Na sensitive to local motion restriction and ionic environment. Reviews highlight that advances in hardware, reconstruction, and sequences are enabling increasingly reliable quantification of sodium concentration and relaxation parameters [110]. Quadrupolar relaxometry further provides additional information on dynamics and local ordering [107].

Applications of ^23^Na illustrate its functional relevance. In breast cancer studies, sodium accumulation has been shown to correlate with tumor progression and treatment response, suggesting its potential as a biomarker beyond conventional imaging [111]. Similar applications exist in neurology and nephrology, including studies of brain, kidney, and ischemic stroke [112,113,114].

In materials science, ^23^Na NMR is particularly relevant in systems containing mobile or partially localized sodium ions, such as solid electrolytes, ionic conductors, and polyelectrolytes. It enables investigation of coordination environments, migration dynamics, and transport mechanisms, which are critical for functional material design [115,116].

In contrast, ^19^F represents a different application paradigm. It is highly favorable in terms of physical properties: spin 1/2, 100% natural abundance, and a high gyromagnetic ratio close to that of protons. This results in high sensitivity and relatively simple interpretation [106,108].

The key advantage of ^19^F in biomedical applications is the negligible endogenous fluorine content in tissues. This means that the detected signal can be directly attributed to introduced tracers or carriers [109,117]. As a result, ^19^F MRI is widely used for cell tracking, inflammation imaging, nanocarrier monitoring, and molecular probe design [117,118,119].

The main limitation of ^19^F is not nuclear physics, but probe chemistry and concentration. To obtain a sufficient signal, materials must contain a large number of equivalent fluorine atoms, favorable relaxation properties, and appropriate stability. Therefore, development of ^19^F MRI is closely linked to the design of fluorinated polymers, nanoemulsions, perfluorocarbons, and related systems [117,119,120].

Reviews emphasize that successful implementation of ^19^F MRI requires optimization of both sequence parameters and chemical probe properties, including spectral complexity, fluorine content, and compatibility with proton imaging [109].

In comparison, ^23^Na is better suited for studying endogenous physiological and ionic processes, while ^19^F is ideal for tracking exogenous agents. ^23^Na suffers from lower sensitivity but provides direct insight into ion transport and homeostasis. ^19^F offers high sensitivity and no background but requires the introduction of fluorinated probes [107,109,117,118,119].

In practice, these nuclei are increasingly used complementarily. Multinuclear imaging studies show that combining proton imaging with sodium, phosphorus, or fluorine signals enables integration of anatomical, functional, and molecular information [106,108,121].

For example, interleaved ^23^Na MRI and ^31^P MRSI at 7 T illustrate the integration of multiple nuclei within a single protocol [121]. Similarly, ^19^F MRI is typically interpreted alongside proton imaging, which provides anatomical reference for fluorine “hot spots” [109,118].

From the perspective of hard and soft materials and biomedical applications, the choice between ^23^Na and ^19^F should be guided by the research question. ^23^Na is appropriate for studying endogenous ion distribution and transport. ^19^F is preferable for tracking labeled systems with high specificity and no background.

Thus, both nuclei extend magnetic resonance beyond proton imaging and demonstrate that, in multinuclear techniques, nucleus selection is fundamentally a functional decision determined by the desired information [106,107,108,109,110,111,112,113,114,115,116,117,118,119,120,121].

## 5. Magnetic Resonance Imaging Beyond Proton MRI

### 5.1. Proton MRI in Materials and Biomedical Research

Proton magnetic resonance imaging (^1^H MRI) remains the fundamental and most widely used form of MR imaging, both in biomedical applications and— increasingly—in materials research. Its privileged position results primarily from the very high abundance of protons in water and organic compounds, the high sensitivity of the ^1^H nucleus, and the well-developed instrumental and methodological infrastructure. Consequently, proton MRI serves as a natural reference point for multinuclear techniques: it provides high-resolution anatomical information, images the distribution of water, fat, and other mobile components, and, when appropriate pulse sequences are used, can also function as a quantitative and functional tool [122,123,124].

In biomedical research, conventional ^1^H MRI is based mainly on contrast arising from proton density and differences in T1 and T2 relaxation times, but contemporary applications of this technique extend far beyond morphological imaging. This is particularly evident in neurology, where proton MRI and related techniques, such as quantitative multiparametric mapping, relaxometry, diffusion imaging, and magnetization transfer, are used to assess tissue microstructure and monitor disease progression. In multiple sclerosis, conventional MRI remains the basis of diagnosis, but quantitative and microstructural parameters are becoming increasingly important, as they may reflect disease activity and tissue damage better than contrast-weighted images alone [125,126]. Thus, proton MRI is no longer merely a tool for detecting lesions, but is becoming a platform for quantitative tissue characterization.

A very important direction in the development of proton MRI is the quantitative assessment of tissue chemical composition based on water and fat signals. A particularly important example is MRI-proton density fat fraction (MRI-PDFF), that is, proton density fat fraction measurement, which has found broad application in studies of the liver, pancreas, muscles, and other organs. This technique enables non-invasive, reproducible, and quantitative assessment of fat accumulation, which is highly relevant in metabolic diseases and therapy monitoring [127,128]. These applications are also expanding beyond the liver, encompassing analysis of fat in skeletal muscles, the pancreas, and bone marrow, where the proton water/fat signal can act as a biomarker of metabolic, degenerative, and inflammatory changes [129,130].

In the musculoskeletal system, proton MRI is particularly valuable because it enables assessment of tissues with very different proton mobility. Conventional sequences image muscles and soft tissues well, whereas the development of ultrashort echo time (UTE) techniques has made it possible to examine components with very short T2 values, such as deep cartilage, tendons, ligaments, and cortical bone. As a result, ^1^H MRI can be used not only for morphological assessment, but also for quantitative evaluation of bound and pore water content, collagen, and the degree of tissue degeneration [131,132]. This is particularly important in cartilage and bone studies, where, after appropriate contrast preparation, the proton signal becomes an indirect marker of matrix composition and structural integrity.

At the same time, functional and molecular proton MRI is developing, based on proton exchange phenomena and magnetization transfer. A good example is amide proton transfer (APT) imaging, one of the CEST techniques, which uses indirect signal amplification through water to detect exchangeable protons in metabolites and macromolecules. In ischemic stroke, APT MRI has proven to be a promising tool for imaging tissue acidosis and pH changes, that is, parameters that are not readily accessible with conventional proton MRI [133,134]. Similar approaches are also being developed in oncology and other diagnostic areas, where proton MRI is acquiring features of molecular imaging without moving away from the ^1^H nucleus.

However, the significance of proton MRI is not limited to clinical biomedicine. In materials research, this technique is particularly valuable when the investigated material contains water, soft phases, or proton-bearing organic components. In such systems, ^1^H MRI allows imaging of the spatial distribution of liquids, swelling, diffusion, functional porosity, and structural changes occurring during material use. This applies especially to hydrogels, biomaterials, tissue scaffolds, polymer composites, and dosimetric materials.

A good example is hydrogels used in three-dimensional radiation dosimetry. Their usefulness results from the fact that local chemical changes induced by irradiation affect the relaxation of water protons and can therefore be read out by MRI as a spatial dose distribution. In this case, proton MRI serves as a tool for non-destructive, three-dimensional characterization of a material responding to a physical stimulus [135]. This is a clear example of the transition from classical medical imaging to the analysis of functional soft materials.

A similar role is played by ^1^H MRI in tissue engineering, where it is used to assess scaffolds and implants. One problem with many biomaterials is low intrinsic contrast in proton imaging, but suitable material modifications can overcome this barrier. In the work of Leon-Chavano et al., a nanoparticle-based ink was developed to enable more precise visualization of tissue scaffolds in MRI, showing that proton imaging can be an effective tool for monitoring the architecture and integration of biomaterials if the material is properly designed for contrast generation. This approach is of practical importance because it enables non-invasive tracking of changes occurring in implants and tissue constructs without the need for destructive analysis [136,137,138,139,140,141,142,143,144,145,146,147,148].

From a broader perspective, proton MRI therefore remains a technique that links two areas of application. In biomedicine, it is the primary tool for anatomical, functional, and increasingly quantitative imaging. In materials science and biomaterials engineering, it enables imaging of the distribution of proton-bearing phases, hydration state, the aqueous microenvironment, and changes occurring in soft and composite materials. It is precisely this versatility that makes ^1^H MRI not only the starting point for multinuclear techniques, but also an independent tool with a very broad informational scope. For this reason, proton imaging should be regarded as a baseline method that, depending on how contrast is prepared, can serve both classical diagnostics and advanced materials analysis [122,123,124,125,126,127,128,129,130,131,132,133,134,135,136].

### 5.2. Sodium Imaging (^23^Na MRI)

Sodium imaging (^23^Na MRI) is an advanced magnetic resonance technique that goes beyond classical proton imaging by enabling direct assessment of sodium ion distribution in tissues. Unlike ^1^H MRI, which mainly reflects water content, ^23^Na MRI provides information on metabolic status and cellular integrity, because sodium concentration is tightly regulated and depends on membrane function and ion transport [137,138].

The contrast mechanism in sodium imaging is based primarily on total tissue sodium concentration (TSC) and its distribution between the intracellular and extracellular spaces. Under pathological conditions, such as cell damage, hypoxia, or metabolic disturbances, intracellular sodium concentration increases, leading to an increase in MRI signal. An important factor affecting contrast is also the quadrupolar nature of the sodium nucleus (spin 3/2), which causes rapid relaxation processes and complex interaction mechanisms with the molecular environment [138,139]. To increase signal selectivity, techniques such as quantum-filtered imaging are used, allowing preferential detection of sodium associated with structures of restricted mobility [140].

One of the best-established areas of application of ^23^Na MRI is kidney disease. This technique enables non-invasive imaging of the sodium concentration gradient between the renal cortex and medulla, which is crucial for the function of the countercurrent mechanism. Experimental studies have shown that in diabetes models, this gradient becomes disrupted, reflecting dysfunction of ion transport and damage to kidney structure [141]. In addition, increased sodium accumulation in tissues observed in chronic kidney disease is associated with the development of hypertension, chronic inflammation, and increased risk of cardiovascular complications, underscoring the potential of this method as a disease biomarker [137,142].

In neuroimaging, ^23^Na MRI enables assessment of regional changes in brain sodium concentration, which are sensitive to disturbances in ion homeostasis and energy metabolism. These changes may reflect cell damage, edema, or alterations in extracellular space volume, making this technique a promising tool in studies of neurological diseases and in monitoring their progression [140]. A similar approach is applied in studies of skeletal muscle and soft tissues, where it is possible to assess changes associated with degenerative processes, inflammation, and disturbances in water–electrolyte balance [143,144].

Despite its important advantages, sodium imaging has significant technical limitations. The most important is the low SNR resulting from the lower sensitivity of the sodium nucleus and its lower concentration in tissues compared with protons. In addition, short relaxation times require the use of specialized sequences with very short echo times, and the achievable spatial resolution remains limited [138,140]. In clinical practice, the use of high magnetic fields and longer acquisition times is often necessary, which limits the availability of this technique. An additional challenge is interpretation of the signal, because it reflects total sodium concentration without unambiguous differentiation between intracellular and extracellular fractions.

In summary, ^23^Na MRI is an important extension of MRI capabilities, enabling assessment of metabolic and ionic processes inaccessible to conventional methods. Despite technological limitations, the development of advanced acquisition and data analysis techniques indicates the growing potential of this method in diagnosis and disease monitoring, particularly in nephrology and neurology [137,138,139].

### 5.3. Fluorine Imaging (^19^F MRI)

Fluorine imaging (^19^F MRI) is a unique extension of magnetic resonance methods beyond classical proton imaging. Its key feature is the ability to generate a signal without biological background. Unlike ^1^H MRI, in which the signal originates mainly from water and lipid protons, fluorine is virtually absent from the human body. Consequently, any recorded ^19^F signal arises exclusively from exogenously administered compounds, which provides very high specificity and unambiguous localization [145,146].

The contrast mechanism in ^19^F MRI is therefore based on direct detection of fluorine nuclei introduced into the body, most often in the form of perfluorocarbons (PFCs) or fluorinated nanoparticles and polymers. These compounds are characterized by a high number of fluorine atoms, which enables their visualization despite the relatively low sensitivity of this technique. The absence of background signal greatly improves contrast utility, because even small amounts of tracer can be detected unambiguously without interference from tissue signal [145,147].

One of the most important applications of ^19^F MRI is tracking contrast agents and labeled cells in vivo. Fluorinated compounds can be used either as contrast carriers or as labels introduced directly into cells, enabling monitoring of their migration, accumulation, and survival over time. This approach is applied, among others, in studies on immunotherapy, inflammatory processes, and regenerative medicine, where transplanted cells or tissue implants can be tracked non-invasively. Owing to the zero-background signal, imaging specificity is very high, exceeding that of classical techniques based on indirect contrast mechanisms [146,148].

An important advantage of ^19^F MRI is also its quantitative potential. Signal intensity is directly proportional to the number of fluorine nuclei in a given voxel, which enables relatively simple and direct quantification of contrast agent concentration. Unlike ^1^H MRI, where the signal depends on many physical parameters (e.g., T1, T2, proton density), ^19^F MRI allows more straightforward quantitative interpretation. This makes it possible to determine the bioavailability, distribution, and pharmacokinetics of administered compounds, which makes this technique particularly attractive in theranostic applications and in therapy monitoring [145,149].

Despite its many advantages, ^19^F MRI remains technologically demanding. The main limitation is low sensitivity resulting from the lower gyromagnetic ratio of fluorine and the limited concentration of tracers in tissues. In practice, this requires the use of high magnetic fields, specialized transmit–receive coils, and prolonged acquisition times. In addition, the design of effective and safe fluorinated compounds is a significant challenge, involving issues of biocompatibility, elimination from the body, and optimization of relaxation properties [147,149].

In summary, ^19^F MRI offers a unique opportunity to obtain background-free signal, precisely track exogenous tracers, and perform their quantitative analysis. These properties make this technique particularly promising in molecular imaging and personalized medicine, although its full clinical implementation still requires further technological development [145,146,147].

## 6. Hardware Used in NMR and MRI

**Figure 4:**
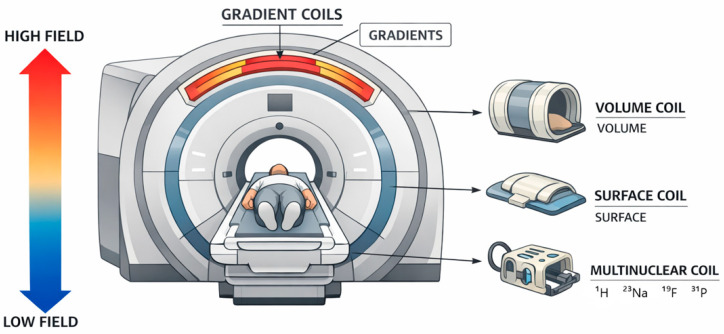
Hardware ecosystem in magnetic resonance. Schematic representation of key components of an MR system, including the main magnetic field (B_0_), gradient coils for spatial encoding, and radiofrequency (RF) coils for signal transmission and reception. Different coil configurations—volume, surface, and multinuclear coils—enable tailored sensitivity and compatibility with various nuclei such as ^1^H, ^23^Na, ^19^F, and ^31^P.

Magnetic resonance relies on a complex hardware ecosystem that determines sensitivity, spatial encoding, and multinuclear capability. Key components include the main magnetic field, gradient systems, and radiofrequency coils, each contributing to signal generation and detection. An overview of this architecture is shown in Figure 4.

### 6.1. Magnetic Field Strength—High-Field and Low-Field Systems

Magnetic field strength is one of the key parameters determining the properties of magnetic resonance imaging, directly affecting SNR, spatial resolution, contrast, and acquisition time. The historical development of MRI was closely linked to the increase in B_0_ field strength, because higher field values enable greater sensitivity and more detailed tissue characterization. At the same time, recent years have seen renewed interest in low-field systems, driven by significant technological advances in scanner design [150,151].

Contemporary low-field MRI systems differ substantially from their earlier counterparts. Their development has been enabled by the introduction of cryogen-free magnets, improvements in RF transmit–receive systems, increased gradient speed and efficiency, and the use of advanced acquisition and reconstruction methods such as parallel imaging, compressed sensing, and artificial intelligence algorithms [150]. As a result, low-field MRI is no longer perceived solely as a quality-based compromise, but has instead emerged as an alternative design approach offering lower cost, greater accessibility, and the possibility of use in settings with limited infrastructure [151,152,153,154].

An important advantage of low-field systems is their lower sensitivity to susceptibility artifacts, as well as improved safety in the presence of implants and medical devices. Consequently, they may find application in specific clinical areas such as bedside imaging, pediatrics, or diagnostics in settings with limited access to advanced medical infrastructure [151,152]. Studies have also shown that, despite lower SNR, reliable and reproducible results can be obtained, for example, in cardiac and musculoskeletal imaging, especially when modern reconstruction methods are used [152,153].

The main limitation of low-field MRI remains the reduced SNR, which affects image quality and forces trade-offs between resolution, scan time, and diagnostic accuracy [151]. In response to these limitations, machine learning-assisted methods are being developed to improve image quality, including super-resolution techniques applied in portable ultra-low-field MRI systems [154]. This indicates that the development of low-field MRI currently depends to a large extent on the synergy between hardware and algorithmic solutions.

By contrast, high-field and ultra-high-field systems (≥7 T) offer a significantly higher signal-to-noise ratio, which translates into improved spatial resolution and contrast and enables more advanced analysis of tissue properties. In neuroimaging in particular, higher fields allow better visualization of fine anatomical structures, microstructural alterations, and pathological processes that are not visible on standard 1.5 T and 3 T systems [155,156]. Studies have shown that 7 T MRI can significantly increase diagnostic sensitivity in areas such as drug-resistant epilepsy, gliomas, and demyelinating diseases [157,158,159,160].

However, the use of ultra-high fields is associated with important technical challenges. These include problems with B_0_ and B_1_ field homogeneity, increased specific absorption rate (SAR), stronger susceptibility artifacts, and greater complexity of RF systems and calibration procedures [155,158]. Consequently, these systems require advanced technical infrastructure and specialized operational expertise, which limits their availability mainly to research institutions and highly specialized clinical centers.

In summary, the choice of magnetic field strength in MRI represents a trade-off between imaging quality and scanner accessibility and cost. Low-field systems are evolving toward greater accessibility and mobility of diagnostic imaging, whereas high-field and ultra-high-field systems remain indispensable in applications requiring the highest spatial resolution and diagnostic sensitivity [150,151,152,153,154,155,156,157,158,159,160].

### 6.2. Coils and Probes

Radiofrequency coils and probes are among the key components of NMR and MRI hardware, because they are responsible for exciting nuclear magnetization and receiving the MR signal. Their geometry, tuning strategy, impedance matching, number of channels, and relationship to the imaged object directly affect B_1_ field homogeneity, SNR, penetration depth, spatial selectivity, and examination safety. Contemporary coil development is aimed not only at improving imaging sensitivity, but also at better anatomical conformity, support for multiple nuclei, SAR reduction, and integration with additional functions such as local shimming or compatibility with multimodal systems [161,162,163,164].

#### 6.2.1. Volume Coils

Volume coils are designed to generate the most homogeneous RF field possible within a relatively large volume encompassing the entire structure of interest or a substantial portion of it. For this reason, they remain the primary solution in applications requiring uniform excitation, particularly in whole-brain imaging, whole-body imaging of small animals, and multinuclear systems where excitation stability is of key importance [151,165]. A classic example is the birdcage coil, whose design continues to be developed for both clinical and preclinical systems. Modifications include changes in geometry, number of rungs, structural asymmetry, and adaptation to unusual spatial or anatomical constraints [165,166,167,168].

In recent years, low-field volume coils designed for open MRI systems have gained particular importance. In such systems, traditional cylindrical geometry is not always optimal, and therefore complex volume resonators and coupled resonator systems are being developed to preserve favorable field distributions in non-standard scanner geometries [161,162]. Work on multimode resonators and stacked configurations shows that, at low field, effective use of available space and improved RF field homogeneity in open systems become just as important as the increase in sensitivity itself [162,169].

At the same time, more specialized volume coil designs are being developed for high-field and preclinical applications. These include high-efficiency ceramic volume coils for small-animal imaging, coils with modified end rings to improve sensitivity in rat brain imaging, and optimized birdcage configurations for ultra-high fields [167,170,171]. In 7 T and higher systems, the design of volume coils must consider not only SNR and B_1_ homogeneity, but also the increase in electromagnetic inhomogeneities and the risk of local heating. For this reason, coil design is increasingly closely linked to advanced electromagnetic simulations [164,172].

#### 6.2.2. Surface Coils

Surface coils are designed primarily for signal reception from regions located close to their conducting elements. Their main advantage is high local SNR, which makes them particularly useful for imaging small anatomical structures, high-resolution studies, and applications in which maximizing sensitivity within a limited field of view is the priority [163,173]. The price for this benefit, however, is a more inhomogeneous sensitivity profile that decreases rapidly with distance from the coil, which requires intensity correction methods and appropriate image reconstruction [174].

Modern approaches to the design of surface coils increasingly rely on the concept of close anatomical conformity. Flexible, stretchable, and self-tuning designs enable better contact with the patient’s body, reduced distance between the coil and tissue, and improved SNR without increasing RF power [163,175]. Particularly interesting are systems based on liquid metals and other deformable conductors, which allow proper tuning to be maintained even when the coil changes shape [163]. These solutions are especially relevant in imaging of joints, limbs, and irregular anatomical regions, where conventional rigid housings limit the quality of fit.

Specialized surface coils are also being developed for preclinical and multimodal applications. Examples include inductively coupled transmit–receive coils for small animals, enabling highly sensitive proton imaging as well as X-nucleus imaging and spectroscopy while preserving access for anesthesia, monitoring, and experimental procedures [173]. In the field of low-field MRI, multimodal surface coils with more complex field distributions are also being developed for new open-system configurations [176]. A separate issue remains optimization of conductor geometry, as illustrated by redesigned meander coils that reduce noise, improve homogeneity, and decrease image artifacts [177].

#### 6.2.3. Multinuclear Coils

The development of MRI and Magnetic Resonance Spectroscopy (MRS) beyond proton signal has driven the emergence of multinuclear coils capable of operating at different Larmor frequencies. These designs must ensure not only proper tuning for several nuclei, but also appropriate channel decoupling, operational stability, and an acceptable compromise between bandwidth, SNR, and system complexity [178,179,180,181]. In practice, both integrated systems and modular designs are used, in which a proton coil operates together with interchangeable elements for X nuclei.

A good example of this approach is provided by systems combining a proton birdcage with interchangeable loops for ^2^H, ^13^C, ^23^Na, and ^31^P, as well as triple-tuned birdcage designs coupled with additional dipole arrays for four-nucleus imaging at 7 T [178,180]. Such an approach enables flexible adaptation of the hardware to a particular experiment without the need to completely rebuild the RF chain. At the same time, it requires advanced baluns, traps, and decoupling solutions that limit common-mode currents and minimize mutual interaction between individual channels [181,182].

Multinuclear capability is particularly important in metabolic and functional studies, where high-resolution proton anatomical data are combined with information from nuclei such as ^13^C, ^23^Na, or ^31^P. Multinuclear designs must therefore accommodate different requirements in excitation, sensitivity, and penetration depth. In preclinical studies, for example, specialized ^13^C/^31^P coils have been shown to effectively support imaging of metabolism and brain energetics in rodents, whereas in clinical systems, there is growing interest in configurations enabling simultaneous or interleaved multinuclear acquisitions [179,183]. As the number of nuclei and channels increases, the design of broadband decoupling systems and high-impedance receivers becomes crucial for limiting SNR degradation and improving frequency separation [182,184].

#### 6.2.4. Development Trends and Design Limitations

Contemporary RF coil and probe design increasingly relies on electromagnetic simulations, which make it possible to predict field distributions, interactions with the sample and conductive elements, and local SAR values even before a prototype is built. This is particularly important in irregular geometries, multinuclear systems, and ultra-high-field scanners [164,172,185]. Both hybrid methods combining volume and surface integral equations, and specialized co-simulation procedures for wireless or non-standard coils, are being developed [161,164].

A second important direction is the integration of coils with additional system functions. Examples include flexible systems combining RF reception with local B_0_ shimming, designs compatible with simultaneous brain stimulation, systems conditioned for patients with deep brain stimulation (DBS) implants, and autonomous impedance-matching solutions [186,187,188,189]. These developments show that the modern MRI coil is no longer merely a passive receiving element, but is becoming an active module that co-determines the quality, safety, and functionality of the entire system.

Despite rapid design progress, coil development still faces a number of limitations. These include trade-offs between homogeneity and local sensitivity, channel decoupling challenges, increased SAR at high fields, dependence of coil characteristics on patient loading, and difficulties in maintaining stable tuning in flexible and deformable designs [163,172,188]. This means that RF coil development remains one of the most dynamic, but also one of the most demanding, areas of NMR and MRI hardware.

## 7. Contrast Mechanisms in NMR and MRI

Contrast mechanisms in NMR and MRI can be divided into two main groups: endogenous contrast, resulting from the natural physicochemical properties of the studied systems, and exogenous contrast, generated after the introduction of external contrast agents. In research and clinical practice, these two approaches are not competitive, but complementary. Endogenous contrast allows assessment of the natural hydration state, ionic composition, and metabolic profile of tissues, whereas exogenous contrast enables increased detection sensitivity and more specific biological and molecular information [190,191,192,193]. A particularly important extension of the latter strategy is represented by targeted contrast agents, designed to recognize specific receptors, cellular structures, or pathological processes [194,195,196,197,198,199,200,201].

### 7.1. Endogenous Contrast

#### 7.1.1. Water

Water is the primary source of endogenous contrast in conventional proton MRI. Its high abundance in biological tissues and the sensitivity of the proton signal to the local molecular environment mean that differences in T1 and T2 relaxation times can be used directly to differentiate structures and pathological states. Water contrast reflects not only total water content, but also the degree of water binding, mobility, and interactions with macromolecules and cell membranes, which gives it both anatomical and functional significance [192,193].

From an interpretative perspective, however, the signal originating from water is relatively nonspecific. Changes in signal intensity may result from edema, inflammation, necrosis, fibrosis, perfusion alterations, or changes in tissue microstructure. Therefore, water contrast alone often requires supplementation with quantitative parameters or other MR techniques [192]. Despite these limitations, it remains the most important and universal source of information in MRI and serves as the reference point for more advanced contrast methods.

#### 7.1.2. Sodium Ions

A second important mechanism of endogenous contrast is the signal arising from sodium ions. ^23^Na MRI provides information unavailable in conventional proton MRI, because it reflects ionic homeostasis, cell membrane integrity, and ion transport activity. Changes in tissue sodium concentration are associated with disturbances in energy metabolism, cellular damage, inflammatory processes, and cancer, and therefore sodium can serve as a sensitive functional biomarker [190,191].

In sodium neuroimaging, it has been shown that ^23^Na MRI can complement conventional proton imaging by better reflecting cellular and metabolic changes, especially in situations where classical MRI shows only secondary consequences of pathology [190]. Similarly, in tissues with a high degree of structural organization, such as the intervertebral disc, quantitative sodium imaging can be used to assess matrix composition and tissue functional state [191].

The limitation of sodium MRI remains its low sensitivity, resulting from the properties of the ^23^Na nucleus, lower signal-to-noise ratio, and short relaxation times. In practice, this means lower spatial resolution and greater hardware demands than in proton imaging [190,191]. Nevertheless, sodium contrast is one of the most important examples of biologically meaningful endogenous multinuclear imaging.

#### 7.1.3. Metabolites

A third group of endogenous contrast sources consists of metabolites detected by MR spectroscopy and spectroscopic imaging methods. In contrast to water contrast, which is based mainly on relaxation, metabolic contrast arises from the presence of specific chemical compounds and their chemical shifts. As a result, MRS provide more direct insight into tissue biochemistry, including energy metabolism, membrane turnover, the presence of neurotransmitters, and changes in synthesis and degradation pathways [192,193].

The importance of metabolic contrast is particularly evident in brain studies, where spectroscopic imaging at high and ultra-high field enables detection of an increasingly broad panel of metabolites and their spatial mapping [193]. From a biological perspective, metabolites associated with choline metabolism, itaconate, and other compounds involved in inflammatory responses and cellular metabolism are of particular interest [194,195,196]. Such signals may serve as markers of functional changes that precede structural changes visible in standard MRI.

However, the limitation of metabolic contrast lies in spectral complexity, signal overlap, and high sensitivity to motion and magnetic field inhomogeneity. For this reason, interpretation of MRS data requires careful standardization of acquisition and processing [192]. Despite these challenges, endogenous metabolites provide a unique layer of information that extends MRI from the level of morphology to the level of function and biochemistry.

### 7.2. Exogenous Contrast

#### 7.2.1. Gadolinium Compounds

The most widely used group of exogenous MRI contrast agents remains gadolinium-based compounds. Their action is based primarily on shortening the T1 relaxation time of nearby water protons, which leads to increased signal intensity in T1-weighted images. As a result, gadolinium chelates are widely used in imaging of inflammatory, neoplastic, and vascular lesions, especially in dynamic techniques such as dynamic contrast-enhanced MRI (DCE-MRI) [197,198].

The advantage of gadolinium agents lies in their high contrast efficiency and well-established clinical role. In diagnostic practice, they enable assessment of perfusion, vascular permeability, blood–brain barrier integrity, and disease activity [197]. At the same time, increasing attention is being paid to the safety limitations of this group of compounds, especially the risk of gadolinium retention and its potential interactions with macromolecules and anionic components of the extracellular matrix [198]. Reports of gadolinium deposition in tissues and long-term biological effects have increased interest in alternative contrast strategies [197,199].

For this reason, the contemporary approach to gadolinium agents is more critical than in the past. Although they remain the basis of many clinical procedures, their use is increasingly considered in the context of a benefit–risk balance, especially where similar information can be obtained using non-contrast methods or other classes of contrast agents [197].

#### 7.2.2. Manganese Compounds

Manganese is an interesting alternative to gadolinium because, as a paramagnetic ion, it can also shorten T1 relaxation, while at the same time exhibiting biological properties relevant to functional imaging. Manganese can enter cells through activity-dependent pathways, which means that it serves not only as a classical contrast enhancer but also as an indicator of cellular function and biological transport [120].

In recent years, the development of manganese-based compounds has focused both on classical contrast applications and on more complex molecular and targeted designs, including manganese porphyrins and systems for cell tracking [200,201]. Experimental studies have shown that manganese can be used to achieve positive T1 contrast while preserving biological usefulness, for example, in monitoring transplanted cells [201].

The limitation of this class of agents, however, remains the potential toxicity of manganese, which depends on dose, chemical form, and exposure time. This is precisely why the translation of many manganese-based solutions into routine clinical practice is slower than in the case of gadolinium contrast agents (GBCAs). Nevertheless, manganese compounds remain an important class of exogenous agents, especially in the context of cellular imaging and the design of new molecular probes [200].

#### 7.2.3. Fluorine-Based Contrast

A distinct category of exogenous contrast is represented by agents based on ^19^F MRI. Their main advantage is the practical absence of background signal in most biological tissues, so the presence of a fluorinated compound can be interpreted very unambiguously. Unlike gadolinium and manganese agents, which modify proton signal indirectly through effects on relaxation, fluorine-based agents generate their own directly detectable signal [202,203,204].

This property makes ^19^F MRI particularly attractive for cell labeling and tracking, immune system monitoring, and imaging of molecular probes and biomedical materials [203,204,205,206,207]. Numerous platforms based on fluorinated nanoparticles, hydrogels, micelles, and stimulus-responsive polymers are being developed, making it possible to tailor their relaxation properties, molecular mobility, and biological specificity [202,203,204,205,206].

The main limitation of fluorine MRI, however, remains its lower sensitivity compared with classical ^1^H MRI and the need to deliver a sufficiently large number of fluorine atoms to the target site. For this reason, the chemical design of the contrast agent is critically important and must simultaneously ensure a high fluorine content, appropriate molecular mobility, good bioavailability, and an acceptable safety profile [204,205]. Despite these challenges, fluorine-based contrast agents are one of the most promising platforms for quantitative and highly specific MR imaging.

### 7.3. Targeted Contrast Agents

#### 7.3.1. Ligand–Receptor Interactions

Targeted contrast agents represent an extension of classical exogenous contrast toward molecular imaging. They are based on the use of specific ligand–receptor interactions, which allow the contrast agent to accumulate preferentially in selected cells, tissues, or pathological niches. Unlike non-specific agents, whose distribution depends mainly on perfusion and vascular permeability, targeted agents are intended to provide information about the expression of specific biological targets [208,209,210].

In practice, the most commonly used targets are receptors overexpressed in tumors, inflammatory cells, or fibrotic tissues. One example is targeting the asialoglycoprotein receptor in liver and fibrosis imaging, where appropriately designed iron oxide nanoparticles showed increased tissue specificity [208]. Similarly, platforms targeting the CXCR4 receptor demonstrate potential in theranostic tumor imaging using MRI/Computed Tomography (CT) methods [209]. The receptor mechanism therefore increases signal selectivity, but its effectiveness depends on the level of target expression, receptor accessibility, and the biological heterogeneity of the lesion.

#### 7.3.2. Chemical Functionalization

Effective performance of targeted agents requires appropriate chemical functionalization. This includes modification of the carrier surface, conjugation of ligands, peptides, antibodies, or small molecules, and adjustment of charge, hydrophilicity, and particle size. These parameters determine pharmacokinetics, the ability to cross biological barriers, immunogenicity, and signal persistence [210,211,212,213,214].

In modern MRI platforms, functionalization is not intended solely to improve receptor binding. Equally important are the enhancement of relaxivity, improvement of biocompatibility, and the combination of several functions in one system, for example, imaging, drug delivery, and monitoring of therapeutic response [211,212,213,214]. As a result, the contrast agent ceases to be a passive signal enhancer and instead becomes an actively engineered molecular platform.

#### 7.3.3. Theranostic Platforms

The most advanced form of targeted agents is represented by theranostic systems, which combine diagnostic and therapeutic functions. In such solutions, the MRI signal serves not only to detect a lesion, but also to control agent delivery, monitor biodistribution, and evaluate treatment efficacy [211,212,213,214]. This applies particularly to magnetic nanoparticles and multifunctional systems used in oncology and central nervous system diseases.

Despite their high translational potential, the development of targeted contrast agents faces significant barriers. These include synthetic complexity, standardization difficulties, biological variability between patients, limited penetration into certain tissues, and the need to demonstrate long-term safety [210,212,214]. For this reason, these agents currently remain primarily an area of intensive preclinical and translational research. At the same time, this group of compounds most clearly illustrates the direction of MRI development from anatomical imaging toward molecular and personalized imaging.

## 8. Comparison of Endogenous and Exogenous Signals

**Figure 5:**
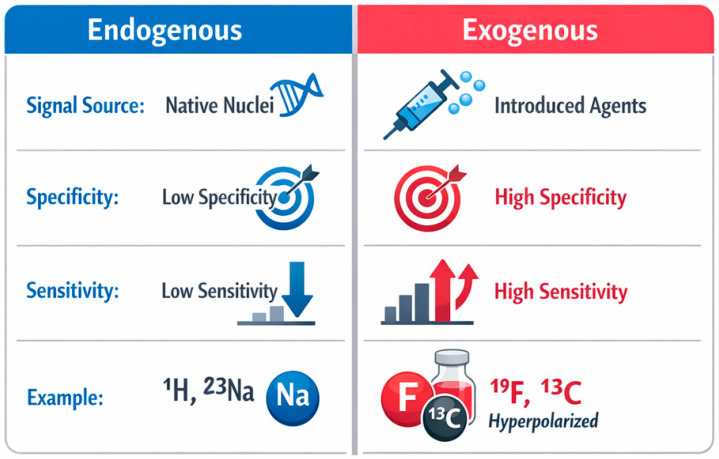
Endogenous vs. exogenous contrast in magnetic resonance. Endogenous contrast originates from native nuclei and intrinsic tissue properties, typically offering limited specificity and sensitivity but broad applicability. In contrast, exogenous approaches rely on administered probes or tracers, enabling higher specificity and sensitivity, particularly in molecular and functional imaging. Representative examples include ^1^H and ^23^Na for endogenous contrast, and ^19^F or hyperpolarized ^13^C for exogenous strategies.

Contrast in magnetic resonance can arise either from intrinsic properties of biological tissues or from externally administered agents. These two paradigms differ fundamentally in terms of specificity, sensitivity, and interpretability. A schematic comparison is presented in Figure 5.

A comparative approach to endogenous and exogenous signals in NMR and MRI is important not only from a technical perspective but also from an interpretative one. Both types of contrast provide biologically useful information, yet they are based on different physical principles and differ in terms of specificity, sensitivity, safety, and methodological requirements. Endogenous signals arise from naturally occurring components of the system, such as water, ions, metabolites, or chemical exchange properties, whereas exogenous signals require the administration of an external contrast agent or molecular probe that alters local relaxation properties or provides an additional MR-detectable signal [215,216,217,218,219].

From an interpretative standpoint, the main advantage of endogenous contrast lies in its close relationship with the physiology and microenvironment of the studied tissue. Techniques based on intrinsic relaxation properties, diffusion, non-contrast perfusion, or proton exchange allow information to be obtained without interfering with the system, which enhances safety and enables repeated longitudinal studies. Examples include perfusion methods based on arterial spin labeling, cardiac parametric mapping, vascular-water-exchange MRI (VEXI) techniques for assessing the blood–brain barrier, and CEST approaches without external contrast agents [217,220,221,222,223]. These methods enable noninvasive evaluation of functional and structural processes, which is particularly valuable in patients requiring repeated examinations or in populations where contrast administration is undesirable.

At the same time, endogenous signals are typically more indirect in their biological meaning. Changes in relaxation times, diffusion, or exchange-based signals may reflect multiple overlapping processes, such as edema, fibrosis, necrosis, metabolic alterations, or microstructural changes. This means that although endogenous contrast is diagnostically valuable, its specificity may be limited, and proper interpretation requires knowledge of the biological context and often the integration of multiple imaging parameters [220,221,222,223,224]. In practice, this represents a shift from simple “lesion detection” toward more complex multiparametric interpretation.

Against this background, exogenous contrast offers a significant advantage in terms of sensitivity and, in many cases, greater diagnostic selectivity. The use of contrast agents allows direct visualization of perfusion, vascular permeability, delayed enhancement, inflammatory activity, or the presence of specific molecular targets. In clinical studies, this advantage is particularly evident in dynamic contrast-enhanced imaging of the breast, liver, or lungs, where early and late uptake patterns provide information that is difficult to obtain with non-contrast methods [224,225,226,227,228]. In many oncological and vascular applications, exogenous contrast agents not only improve lesion detectability but also enhance assessment of their biological nature and treatment response [225,227,228].

However, an exogenous signal is not always the optimal solution. Its use requires intravenous administration, is dependent on pharmacokinetics, is influenced by renal function, and carries a potential risk of adverse effects. This is particularly relevant for gadolinium-based agents, for which concerns persist regarding metal retention, transmetallation, and long-term safety [229,230]. In addition, exogenous signals can be strongly dependent on acquisition timing, dose, administration protocol, and analysis model, which increases methodological complexity and complicates inter-center comparability [226,228].

A comparison of both strategies therefore reveals a fundamental distinction: endogenous contrast is generally more physiological, safer, and better suited for repeated studies, whereas exogenous contrast more often provides higher sensitivity, improved lesion detectability, and the possibility of molecular targeting [216,217,219]. In practice, the choice between them should not be treated as binary, but rather as dependent on the research question. If the goal is to assess global tissue properties, monitor changes over time, or study patients with contraindications to contrast agents, endogenous techniques are particularly advantageous. If, on the other hand, the priority is detection of small lesions, perfusion analysis, or obtaining information about a specific biomarker, exogenous methods retain a clear advantage [221,224,227].

The criteria for selecting an appropriate strategy should therefore include several levels. The first is the biological question: whether the study aims to reveal intrinsic tissue properties or requires selective enhancement of a specific process. The second is the desired level of specificity: endogenous contrast more often provides integrated information, whereas exogenous contrast can be more targeted, especially in the case of activatable probes, nanoparticles, or theranostic platforms [218,219,231,232]. The third criterion involves practical constraints, such as patient safety, the possibility of repeated examinations, equipment availability, cost, and acquisition time. The fourth concerns data interpretation: endogenous techniques typically require more cautious linkage between signal and biological mechanism, whereas exogenous techniques require better control of pharmacokinetics and agent–environment interactions [220,229,230].

An increasingly important trend is the hybrid approach, combining both types of signal. In many modern diagnostic strategies, endogenous contrast serves as a reference for tissue structure and function, while exogenous contrast provides an additional layer of molecular or hemodynamic information. This approach is being developed both in conventional MRI and in multimodal systems, as well as in the design of intelligent probes that respond to stimuli present in the pathological microenvironment [218,231,233]. For this reason, the future of MR imaging will likely not involve replacing one type of contrast with another, but rather their increasingly deliberate integration.

In summary, endogenous and exogenous signals should be regarded as two complementary ways of obtaining information about a studied system. Endogenous contrast provides greater naturalness of measurement, safety, and the possibility of multiparametric assessment of physiology, but may be less specific. Exogenous contrast increases sensitivity and expands the scope of molecular imaging, but at the cost of greater methodological complexity and potential safety limitations. The choice of research strategy should therefore be guided not by preference for a given technique, but by alignment with the biological, clinical, and analytical objectives of the study [216,217,224,229].

## 9. Opportunities and Challenges in Multinuclear NMR and MRI

Multinuclear NMR and MRI are currently emerging as one of the most important extensions of conventional proton-based imaging. Their value lies in the ability to provide information complementary to the ^1^H signal, including ionic homeostasis, high-energy metabolism, mineral composition, membrane turnover, and the distribution of selected labeled compounds. As a result, multinuclear techniques enable a shift from predominantly morphological imaging toward functional, metabolic, and molecular imaging [234,235].

At the same time, their broader implementation remains limited by sensitivity constraints, interpretative complexity, hardware requirements, and the lack of full methodological standardization [236,237]. The choice of nucleus in MR experiments reflects a trade-off between sensitivity, biological relevance, and technical feasibility, as summarized in Table 3.

### 9.1. Sensitivity and Signal-to-Noise Ratio

The most important practical limitation of multinuclear NMR and MRI remains sensitivity. Compared to proton imaging, many nuclei such as ^23^Na, ^31^P, ^13^C, or ^7^Li generate weaker signals due to less favorable magnetic properties, lower tissue concentrations, or shorter effective relaxation times. As a result, multinuclear studies are particularly sensitive to reductions in SNR, forcing a compromise between spatial resolution, acquisition time, and quantitative accuracy [234,236,238].

This limitation is well illustrated by sodium imaging, where the biological relevance of the signal is high, but image quality has historically constrained clinical applications. Only recent advances in multinuclear coil design, high-field systems, sequence optimization, and reconstruction techniques have enabled ^23^Na MRI to approach practical translational use [238,239,240]. In brain studies, specialized proton–sodium coil systems at 7 T have improved local SNR and excitation homogeneity, directly enhancing imaging performance [240].

Sensitivity improvements are achieved not only through hardware but also through novel signal enhancement and data processing strategies. Approaches such as hyperpolarization and parahydrogen-based methods can significantly increase the detectability of heteronuclear signals [241]. In parallel, machine learning-based denoising and AI-assisted reconstruction methods are increasingly used to improve apparent image quality. However, these approaches must be applied cautiously, as improvements in visual quality do not always correspond to improved quantitative accuracy.

In practice, sensitivity is not merely a technical parameter but a key determinant of whether a multinuclear technique can be reliably applied for clinical or biological interpretation. Limited SNR remains one of the main reasons why many promising approaches are still confined to preclinical research or specialized centers [236,242].

### 9.2. Data Interpretation in Heterogeneous Systems

A second major challenge lies in the interpretation of data obtained from biologically and materially heterogeneous systems. In real tissues, MR signals typically represent a mixture of multiple compartments, chemical states, and overlapping physiological processes. This complexity becomes even more pronounced in multinuclear imaging, where signals are often more biologically specific but also more sensitive to local microenvironmental variability [243,244,245].

In brain tumors, for example, simultaneous assessment of sodium, diffusion, and perfusion provides insight into different aspects of tumor biology. However, these parameters are not linearly related and vary across tumor regions, requiring interpretation that accounts for both intratumoral heterogeneity and interpatient variability [245]. Similarly, in studies of muscle, brain, or bone, signals from different nuclei should be interpreted as complementary rather than independent markers of biological processes [237,242,246,247].

Spectroscopic data further illustrate this complexity. Even well-resolved MRS spectra represent a superposition of signals from different cell types, metabolic states, and microenvironments. While ultrahigh-field systems improve spectral resolution, they also increase the risk of overinterpreting subtle differences in metabolite concentrations [220,225].

As a result, interpretation of multinuclear data increasingly relies on integrated approaches combining proton MRI, MRS, quantitative imaging, and computational analysis. While this enhances biological relevance, it also increases methodological complexity and limits straightforward comparison between studies.

### 9.3. Technical and Methodological Limitations

The development of multinuclear NMR and MRI is strongly influenced by hardware constraints. Each additional nucleus requires dedicated RF coils, tuning and matching procedures, often separate pulse sequences, and additional calibration steps. This increases experimental complexity, prolongs setup time, and limits availability outside specialized research centers [236,240]. From a translational perspective, these technical requirements also create substantial economic and practical barriers. Advanced multinuclear MR often depends on expensive high- or ultrahigh-field scanners, dedicated multinuclear RF coils, specialized reconstruction software, trained personnel, and longer examination protocols. These requirements are difficult to meet in many local or regional hospitals, where scanner time, reimbursement structures, service contracts, staff training, and compatibility with routine clinical workflows strongly influence feasibility. As a result, many multinuclear techniques remain concentrated in research institutions or highly specialized clinical centers rather than being broadly implemented in routine hospital diagnostics.

Even when appropriate hardware is available, challenges remain in maintaining magnetic field homogeneity, frequency stability, and effective shimming—particularly at lower field strengths or in complex sample geometries. Localized shimming approaches have been shown to mitigate some of these limitations and enable simultaneous multinuclear spectroscopy under certain conditions [237].

Methodological limitations also affect acquisition and data analysis. Multinuclear techniques often require longer acquisition times, increasing sensitivity to motion, physiological variability, and system drift. In addition, the lack of standardized analysis protocols complicates cross-study comparisons. This issue is amplified in multinuclear imaging due to lower intrinsic SNR and higher sensitivity to processing choices [248,249,250].

Further challenges arise in multiparametric and multimodal imaging. Although combining multiple nuclei and contrasts increases information content, it also introduces issues related to spatial resolution mismatch, geometric distortions, temporal misalignment, and image registration errors. Without rigorous quality control, the addition of more parameters does not necessarily translate into improved diagnostic performance [245,251,252].

Finally, many multinuclear techniques operate at the limits of clinically available hardware. Ultrahigh-field systems improve SNR and spectral resolution but introduce additional challenges related to field inhomogeneity, susceptibility artifacts, and safety considerations, while also limiting accessibility [242,253].

### 9.4. Future Directions

Despite these challenges, the future of multinuclear NMR and MRI remains highly promising. One key direction is the integration of multiple nuclei with conventional proton imaging within a single examination. Interleaved acquisition strategies and multiparametric protocols can reduce scan time while combining structural, functional, and metabolic information [239]. Such approaches are particularly relevant in neuro-oncology, metabolic disorders, muscle imaging, and therapy monitoring [242,245,246].

Another important direction is the development of advanced computational tools. Artificial intelligence, model-based reconstruction, denoising, segmentation, and multidimensional analysis are expected to play an increasingly important role in improving image quality and extracting biologically meaningful information from low-SNR data [238,254]. This is especially critical for techniques such as ^23^Na MRI, hyperpolarized ^13^C imaging, and gas imaging.

Progress is also driven by advances in RF technology, including specialized coils, multi-tuned systems, and high-channel-count receiver arrays. These developments improve sensitivity and facilitate more efficient multinuclear acquisition, gradually bridging the gap between research and clinical applications [240,255,256,257]. In parallel, emerging polarization strategies may significantly expand the range of detectable processes [241].

Finally, biological and clinical validation is becoming increasingly important. The future of multinuclear MRI depends not only on demonstrating technical feasibility but also on proving that these techniques provide clinically actionable information that improves diagnosis, treatment planning, or therapy monitoring [233,236,242].

## 10. Conclusions

Multinuclear NMR and MRI fundamentally redefine the scope of magnetic resonance by extending it beyond the traditional proton-centric paradigm. While ^1^H MRI has established itself as an indispensable tool for high-resolution anatomical and functional imaging, its inherent limitations in chemical specificity and molecular selectivity necessitate complementary approaches. The integration of heteronuclear techniques encompassing nuclei such as ^13^C, ^31^P, ^23^Na, and ^19^F opens access to previously inaccessible dimensions of information, including metabolic fluxes, ionic regulation, membrane dynamics, and the distribution of targeted molecular probes. In this sense, multinuclear magnetic resonance represents not merely a technical extension, but a conceptual shift toward a more comprehensive and mechanistically grounded understanding of complex systems.

A central theme emerging from this work is that the informational value of magnetic resonance is intrinsically nucleus-dependent. Each nucleus provides a distinct window into the system under investigation, governed by its magnetic properties, natural abundance, and biological or material relevance. As highlighted throughout this review, the selection of a given nucleus is not arbitrary but reflects a balance between sensitivity, specificity, and experimental feasibility. Proton imaging offers unmatched robustness and spatial resolution, serving as the structural and methodological backbone of MR. In contrast, heteronuclear modalities provide targeted insights that are often more directly linked to specific physicochemical or biological processes, albeit at the cost of reduced signal strength and increased technical complexity. The true strength of multinuclear MR therefore lies not in replacing proton imaging but in its strategic integration with it.

Despite its considerable promise, the widespread adoption of multinuclear NMR and MRI remains constrained by several interrelated challenges. Foremost among these is limited sensitivity, which directly impacts signal-to-noise ratio and imposes trade-offs between spatial resolution, acquisition time, and quantitative accuracy. This limitation is particularly pronounced for nuclei with low in vivo concentrations or unfavorable relaxation properties. In parallel, the interpretation of multinuclear data is complicated by the inherent heterogeneity of biological and material systems, where observed signals often represent composite contributions from multiple compartments, states, and dynamic processes. These challenges are further amplified by the technical demands associated with multinuclear hardware, including specialized RF coils, frequency tuning, calibration procedures, and the need for robust and reproducible acquisition protocols.

Nevertheless, the trajectory of current research strongly suggests that many of these limitations are being progressively mitigated. Advances in ultrahigh-field MRI, high-performance gradient systems, and dedicated multinuclear RF architectures are steadily improving sensitivity and spatial resolution. At the same time, emerging techniques such as hyperpolarization and parahydrogen-based signal enhancement offer the potential for orders-of-magnitude increases in detectable signal, particularly in metabolic imaging. Equally transformative is the rapid development of computational methods, including artificial intelligence-driven reconstruction, denoising, and multiparametric data integration, which are redefining the limits of what can be extracted from inherently low-SNR datasets. Together, these developments point toward a future in which hardware and software advances act synergistically to overcome fundamental physical constraints.

An equally important direction is the growing emphasis on multimodal and multiparametric integration. The combination of proton imaging with heteronuclear MRI and MRS within unified acquisition frameworks enables the simultaneous assessment of structure, function, and molecular composition. Such approaches are particularly promising in areas such as neuro-oncology, metabolic disorders, musculoskeletal imaging, and biomaterials research, where complex pathophysiology cannot be adequately captured by a single contrast mechanism. In this context, multinuclear MR should be viewed not as a collection of isolated techniques, but as part of an integrated imaging ecosystem capable of capturing multiple layers of biological organization.

From a translational perspective, the ultimate impact of multinuclear magnetic resonance will depend on its ability to deliver clinically and scientifically actionable information. Demonstrating clear added value over established proton-based methods whether in terms of earlier detection, improved specificity, or enhanced monitoring of therapeutic response will be essential for broader adoption. This, in turn, requires rigorous validation, standardization of acquisition and analysis protocols, and careful consideration of reproducibility across platforms and institutions. The transition from proof-of-concept studies to routine application thus represents a critical phase in the maturation of the field.

In conclusion, multinuclear NMR and MRI constitute a powerful and rapidly evolving framework for probing the structure–function relationships of complex materials and biological systems across multiple scales. By enabling access to complementary physicochemical and biochemical information, these techniques significantly expand the analytical capabilities of magnetic resonance. While important technical and interpretative challenges remain, ongoing advances in instrumentation, methodology, and data science are steadily transforming multinuclear MR from a specialized research tool into a versatile platform with broad applicability. Its continued development is likely to play a pivotal role in the progression toward more quantitative, mechanism-driven, and ultimately personalized approaches in both materials science and biomedical imaging.

## Figures and Tables

**Table 1:** Approximate Larmor frequencies of selected nuclei at 3 T.

Nucleus	Spin	Natural Abundance (%)	γ/2π (MHz/T)	Approximate Larmor Frequency at 3T (MHz)
^1^H	1/2	99.98	42.58	127.7
^19^F	1/2	100	40.05	120.2
^31^P	1/2	100	17.24	51.7
^23^Na	3/2	100	11.26	33.8
^13^C	1/2	1.1	10.71	32.1

Values are approximate and were calculated for B_0_ = 3 T according to Equation (1).

**Table 2:** Magnetic resonance in different states of matter. Summary of the main properties, commonly used techniques, and associated limitations for MR in gases, liquids, and solids.

Phase	Main Characteristics	Techniques	Challenges
Gas	Low density, weak signal	Hyperpolarization (e.g., ^129^Xe, ^3^He MRI)	Low SNR, short polarization lifetime
Liquid	Rapid molecular motion, diffusion	Gradient-based MRI, diffusion imaging	Motion artifacts, signal averaging
Solid	Restricted motion, strong interactions	MAS, ssNMR	Line broadening, short T2

**Table 3:** Main technical and methodological challenges in multinuclear magnetic resonance.

Challenge	Cause	Potential Solutions
Low sensitivity	Low γ, low concentration, fast relaxation	Higher field strength, optimized coils, hyperpolarization
Hardware limitations	Need for multinuclear RF systems	Dedicated coils, broadband systems
Short relaxation times	Quadrupolar interactions, molecular environment	Fast sequences, ultrashort echo time (UTE)
Quantification difficulties	Calibration issues, variable relaxation	Standardization, phantom-based calibration
Long acquisition times	Low SNR	Signal averaging, acceleration techniques

## Data Availability

No new data were created or analyzed in this study. Data sharing is not applicable to this article.

## Abbreviations

The following abbreviations are used in this manuscript:

2D	two-dimensional
APIs	active pharmaceutical ingredients
APT	amide proton transfer
CEST	chemical exchange saturation transfer
CPMAS	cross-polarization magic angle spinning
CT	computed tomography
DBS	deep brain stimulation
DCE-MRI	dynamic contrast-enhanced MRI
d-DNP	dissolution-dynamic nuclear polarization
DFT	density functional theory
GBCAs	gadolinium contrast agents
HY	hyperpolarization
MAS	magic angle spinning
MOFs	metal–organic frameworks
MRI	magnetic resonance imaging
MRI-PDFF	MRI-proton density fat fraction
MRS	magnetic resonance spectroscopy
MRSI	MR spectroscopic imaging
NMR	nuclear magnetic resonance
PFCs	perfluorocarbons
PFG	pulsed-field gradient
Photo-CIDNP	photochemically induced dynamic nuclear polarization
REDOR	rotational-echo double resonance
SAR	specific absorption rate
SNR	signal-to-noise ratio
ssNMR	solid-state NMR
TSC	tissue sodium concentration
UHMWPE	ultrahigh-molecular-weight polyethylene
UTE	ultrashort echo time
VEXI	vascular-water-exchange MRI
